# Tamoxifen prolongs survival and alleviates symptoms in mice with fatal X-linked myotubular myopathy

**DOI:** 10.1038/s41467-018-07058-4

**Published:** 2018-11-19

**Authors:** Elinam Gayi, Laurence A. Neff, Xènia Massana Muñoz, Hesham M. Ismail, Marta Sierra, Thomas Mercier, Laurent A. Décosterd, Jocelyn Laporte, Belinda S. Cowling, Olivier M. Dorchies, Leonardo Scapozza

**Affiliations:** 1 Pharmaceutical Biochemistry Group, School of Pharmaceutical Sciences, University of Lausanne, University of Geneva, CMU 5-6, Rue Michel-Servet 1, Geneva, 1211 Switzerland; 2 Department of Translational Medicine and Neurogenetics, Institut de Génétique et de Biologie Moléculaire et Cellulaire (IGBMC), Illkirch, 67404 France; 3 Centre National de la Recherche Scientifique (CNRS), UMR7104, Illkirch, 67404 France; 4 Institut National de la Santé et de la Recherche Médicale (INSERM), U1258, Illkirch, 67404 France; 5 Université de Strasbourg, Illkirch, 67404 France; 6 Division and Laboratory of Clinical Pharmacology, Service of Biomedicine, Department of Laboratories, Lausanne University Hospital, Lausanne, 1011 Switzerland

## Abstract

X-linked myotubular myopathy (XLMTM, also known as XLCNM) is a severe congenital muscular disorder due to mutations in the myotubularin gene, *MTM1*. It is characterized by generalized hypotonia, leading to neonatal death of most patients. No specific treatment exists. Here, we show that tamoxifen, a well-known drug used against breast cancer, rescues the phenotype of *Mtm1*-deficient mice. Tamoxifen increases lifespan several-fold while improving overall motor function and preventing disease progression including lower limb paralysis. Tamoxifen corrects functional, histological and molecular hallmarks of XLMTM, with improved force output, myonuclei positioning, myofibrillar structure, triad number, and excitation-contraction coupling. Tamoxifen normalizes the expression level of the XLMTM disease modifiers DNM2 and PI3KC2B, likely contributing to the phenotypic rescue. Our findings demonstrate that tamoxifen is a promising candidate for clinical evaluation in XLMTM patients.

## Abstract (web summary)

X-linked myotubular myopathy (XLMTM) is a severe muscle disease with no effective treatment. Here, the authors show that tamoxifen, a drug used to treat breast cancer, rescues the pathology in a mouse model of the disease, at least in part by normalizing expression of the disease modifier proteins DNM2 and BIN1

## Introduction

X-linked centronuclear myopathy (XLCNM; OMIM #310400), the most severe form of centronuclear myopathy (CNM), affects ~1 in 50,000 males^1^. XLCNM is commonly known as X-linked myotubular myopathy (XLMTM) as characteristic histological features include small-caliber myofibers and centrally located nuclei resembling myotubes^2,3^. XLMTM patients present with profound and generalized muscle weakness from birth. Most XLMTM male patients die in the first 2 years of life; some develop a milder form and may survive into adulthood while female carriers display a more heterogeneous disease^1,4–6^. XLMTM is due to the lack of myotubularin (MTM1), a lipid phosphatase that dephosphorylates phosphatidylinositol 3-phosphate (PtdIns(3)P) and PtdIns(3,5)P_2_ into PtdIns and PtdIns(5)P^7–9^. Imbalance in these lipids impairs membrane trafficking, nuclei positioning, and t-tubule organization^10^.

No specific treatment exists for XLMTM. AAV-mediated *MTM1* replacement therapy showed great promise in murine and canine models and has reached clinical stage (ClinicalTrials.gov Identifier: NCT03199469)^11–14^. Down-regulating dynamin-2 (DNM2), a protein mutated in autosomal forms of CNM, is another promising approach to rescue several CNM forms^15–17^. The development of these innovative therapies, however, may be hampered by technical and safety issues and be further complicated by cost issues in case of clinical efficacy. By contrast, repurposing well-known drugs might offer time- and cost-effective therapeutic options^18^.

Tamoxifen has been used for almost 40 years to treat estrogen receptor-positive breast cancers in both women and men^19–21^ and has been tried in a variety of other disorders^22–27^. Importantly, tamoxifen proved safe in pediatric patients^28–35^. Our earlier published^36,37^ and unpublished findings that tamoxifen potently counteracted the symptoms in a mouse model of Duchenne muscular dystrophy (DMD) have been translated into compassionate use and a phase 3 clinical trial in underway on DMD boys (ClinicalTrials.gov Identifiers NCT02835079 and NCT03354039, respectively).

We thus thought to evaluate tamoxifen in *Mtm1*^−/y^ mice, a validated model of XLMTM^15,38–40^. In the present work, we demonstrate that oral tamoxifen given to pups from weaning onwards significantly corrects functional, histological, and molecular hallmarks of the disease, resulting in a remarkable enhancement in survival. Tamoxifen is the first EMA- and FDA-approved drug to show such a promising therapeutic potential for patients with XLMTM.

## Results

### Effects of oral tamoxifen treatments on food and drug intake

*Mtm1*^−/y^ mice and wild type (WT) littermates were weaned on post-natal day 23 ± 1, slightly after disease onset, at which time they were given a control diet or pellets supplemented with tamoxifen citrate (equivalent to 30, 10, and 3 mg tamoxifen free base per kg of diet). Food intake was similar on control and high-tamoxifen diets throughout the study time for *Mtm1*^−/y^ and wild type mice (Supplementary Figure 1). On the highest dose (30 mg kg^−1^ of diet), tamoxifen intake was approximately 6, 5, and 4 mg kg^−1^ d^−1^ in treated mice aged 42, 84, and 200 days, respectively. The levels of tamoxifen and major metabolites in plasma and leg muscle tissue were consistent with levels found previously in treated dystrophic mice^36^ (Supplementary Table 1).

### Tamoxifen improves life span and slows disease progression

**Fig. 1:**
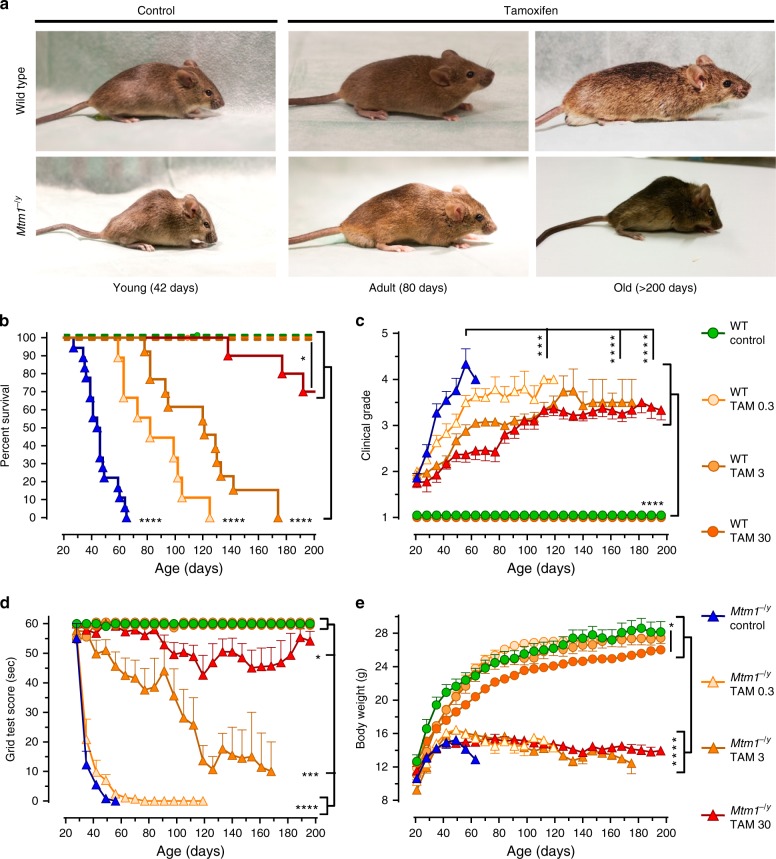
Tamoxifen rescues the phenotype of XLMTM mice. Life-long oral tamoxifen delays disease progression in XLMTM mice. Wild type (WT) and *Mtm1*^−/y^ mice were fed control or tamoxifen (TAM)-supplemented diets from weaning onward. **a** Photographs of WT and *Mtm1*^−/y^ mice, illustrating disease severity in young untreated mice and protection conferred by tamoxifen in adult and old mice. **b** Kaplan–Meier curves showing the effects of treatments on mouse survival. Early death of untreated *Mtm1*^−/y^ mice contrasts with prolongation of life span in tamoxifen-treated littermates. Data are from 8 to 18 mice per group. *P ≤ 0.05; ****P ≤ 0.0001. Log-rank (Mantel–Cox) test. **c** Disease progression was assessed three times per week using a 5-grades clinical scale: muscle function was scored as 1 (normal function of hind limbs), 2 (difficulty in spreading toes), 3 (evident weakness in legs), 4 (paralysis of one hind limb), or 5 (complete paralysis of both legs). WT mice had a clinical score of 1 (normal) throughout the study. Untreated *Mtm1*^−/y^ mice quickly reached a high clinical grade, whereas disease progressed much slower in tamoxifen-treated littermates. **d** Mouse motor function was assessed weekly via a horizontal grid-hanging test. The score of untreated *Mtm1*^−/y^ mice declined quickly. Tamoxifen preserved motor function of *Mtm1*^−/y^ mice close to WT values. **e** Mouse body weight was recorded 3 times per week. Tamoxifen affected the growth of WT but not of *Mtm1*^−/y^ mice. *Mtm1*^−/y^ mice remained smaller than WT mice throughout. **b**–**e** Symbols and TAM doses (0.3, 3, and 30 mg kg^−1^ of diet) are shown on the right-hand side. **c**, **d**, **e** Data represent the mean ± s.e.m. of 8 to 18 mice as defined in **a**. *P ≤ 0.05; **P ≤ 0.01; ****P ≤ 0.0001; ns non-significant. One-way ANOVA with Fisher’s least significance difference (LSD) post-test

Untreated *Mtm1*^−/y^ mice showed early hind limb paralysis with rapid progression of muscle weakness to the trunk and forelimbs, resulting in marked kyphosis, frailty (Fig. 1a) and premature death at a median age of 45 days (Fig. 1b). By contrast, all doses of tamoxifen extended the life span of *Mtm1*^−/y^ mice, which on the highest dose, reached a median age of 290 days. Shortly before dying, all untreated *Mtm1*^−/y^ mice showed paralyzed hindlimbs and dragging feet. By contrast, adult-treated mice remained mobile and retained ability to rear and to climb onto objects using both forelimbs and hindlimbs (Supplementary Movies 1–4). Remarkably, one *Mtm1*^−/y^ mouse on high tamoxifen reached 464 days of age. Consistent with tamoxifen-mediated enhanced survival, disease progression was significantly delayed in treated *Mtm1*^−/y^ mice (Fig. 1c), which performed almost as well as WT mice in an anti-gravity assay assessing whole-body force (Fig. 1d). Old *Mtm1*^−/y^ mice on high tamoxifen diet presented less clinical features of disease and relatively spared upper body (Fig. 1a), suggesting slower retrograde disease progression. This was illustrated by the ability of the treated mice to climb onto objects using their forelimbs, preserved neck and head control, and relatively large thoracic cage suggestive of spared trunk muscles (Supplementary Movies 3, 4; Fig. 1a). In spite of overwhelming protection from the disease, tamoxifen-treated *Mtm1*^−/y^ mice remained small (Fig. 1e). Similarly, tamoxifen-treated WT mice were smaller than their untreated counterparts, partly due to smaller muscles (Supplementary Tables 2, 3), and presumably also because of reduced amount of white adipose tissue, which was evident upon dissection and similar to our observation in dystrophic mice reported earlier^36^.

In order to examine the impact of tamoxifen on muscle mechanical properties, muscle structure and molecular adaptation underpinning tamoxifen-mediated protection, we next treated mice for various periods with optimal tamoxifen dosing (30 mg kg^−1^ of diet).

### Tamoxifen improves the size of leg muscles and diaphragm

Compared with WT mice, most locomotor muscles examined in *Mtm1*^−/y^ mice showed severe atrophy at D42 (Supplementary Table 2, 3). That atrophy was partly prevented by tamoxifen even after normalization for improved body weight (from +10% in the quadriceps to +18% in the gastrocnemius). Overall, the effect of tamoxifen at increasing muscle size of *Mtm1*^−/y^ mice persisted until D210. The diaphragm of *Mtm1*^−/y^ mice showed a distinct response to tamoxifen: although the diaphragm was atrophic at D42 and D84, its absolute size doubled between D84 and D210. As a consequence of changes in body weight, the relative size of the diaphragm was gradually bigger over time (from 67 to 198% of wild-type counterparts at D42 and D210, respectively).

### Tamoxifen improves the strength and features of leg muscles

Muscle weakness is a key pathological feature of XLMTM patients that is also prominent in the *Mtm1*^−/y^ murine model. We investigated if and to what extent tamoxifen ameliorated the force generated by the triceps surae (hereafter referred to as triceps), a large muscle group of the lower leg that makes up most of the calf volume and contains gastrocnemius, plantaris, and soleus muscles.

**Fig. 2:**
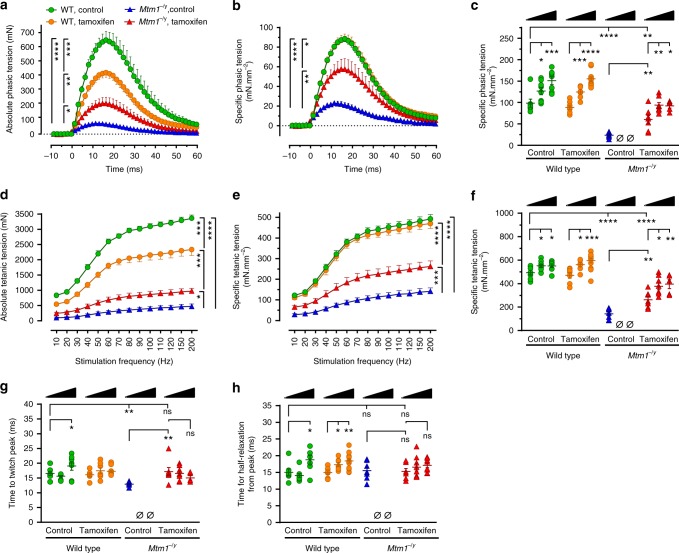
Tamoxifen augments the strength of leg muscles of XLMTM mice. Electrically evoked triceps contractions were recorded under isometric conditions and contractile features analyzed in 42, 84, and 210-day-old mice fed placebo or tamoxifen-supplemented (30 mg kg^−1^) diets. **a** Average traces showing absolute phasic (twitch) contraction of the triceps at 42 days (D42). Tamoxifen significantly enhanced the force (tension) in *Mtm1*^−/y^ mice. **b** Specific phasic force (see Online Methods for details) of D42 *Mtm1*^−/y^ mice augmented with tamoxifen. **c** Specific phasic force of *Mtm1*^−/y^ mice increased with tamoxifen at all examined ages. **d** Average traces showing triceps absolute force-frequency curves at D42. Tamoxifen more than doubled the tetanic force of *Mtm1*^−/y^ mice. **e** Specific force of *Mtm1*^−/y^ mice markedly augmented with tamoxifen at all stimulation frequencies. **f** Specific tetanic force of *Mtm1*^−/y^ mice was considerably improved at all studied ages. In triceps examined after various treatment durations, tamoxifen rescued the impaired time to twitch peak of *Mtm1*^−/y^ mice (**g**) but had no impact on the time required for half-relaxation from peak (**h**). D42 panels: WT wild type; legend in **a** stands for **a**, **b**, **d**, **e**; data represent the mean ± s.e.m. of *n* = 7 triceps per group. **c**, **f**, **g**, **h**: black triangles illustrate increasing age (42–84 and 210 days) within each treatment group; columns from left to right: data represent the mean ± s.e.m. of 7; 8; 7; 7; 7; 13; 7; 0; 0; 7; 8; 6 triceps, respectively. *P ≤ 0.05; **P ≤ 0.01; ***P ≤ 0.001; ****P ≤ 0.0001; ns non-significant, Ø no surviving mice. One-way ANOVA followed by Fisher’s LSD post-test

We first analyzed phasic (also known as twitch) force at D42, at which time a substantial proportion of untreated *Mtm1*^−/y^ mice survived. The absolute phasic force of untreated *Mtm1*^−/y^ mice was highly compromised, representing only 17.3% of that of untreated wild-type mice. Tamoxifen significantly enhanced the absolute force of *Mtm1*^−/y^ mice almost threefold (Fig. 2a). The absolute phasic traces were normalized to the triceps cross-sectional area in order to express the specific phasic tension, i.e., the force generated per unit of muscle cross-section (Fig. 2b). This revealed that tamoxifen markedly augmented the specific phasic force of *Mtm1*^−/y^ mice (+155%), reaching 61% of the force developed by WT mice. We then compared the specific phasic force at D42, D84, and D210 (Fig. 2c). Tamoxifen considerably increased this parameter in *Mtm1*^−/y^ mice at all ages studied compared to untreated *Mtm1*^−/y^ mice at D42 (Fig. 2c). Functional benefits culminated to 74.1% of WT values at D84 and showed only marginal decrease at D210.

Then, the tetanic forces were determined from force–frequency curves. Tamoxifen more than doubled (2.1-fold increase) the absolute tetanic force of *Mtm1*^−/y^ mice at D42, which, after correction for muscle size, reached 53.2% of WT values (Fig. 2d, e). Similar to phasic force, tamoxifen remarkably increased the specific tetanic force of *Mtm1*^−/y^ mice at all ages studied (Fig. 2f). Maximum benefits were found at D210, when tetanic force reached 71.7% of age-matched wild type values (68.2% at D84).

Further analysis of the phasic traces revealed a significant reduction in the time required by the triceps of untreated *Mtm1*^−/y^ mice for reaching twitch peak force, indicative of impaired excitation–contraction coupling (Fig. 2g). Tamoxifen fully restored the time to twitch peak to normal values at all ages. Tamoxifen did not significantly alter the time for half-relaxation from twitch peak at D42 (indicative of SERCA activity and kinetics of inactivation of actin–myosin cross-bridges) (Fig. 2h). Nevertheless, this feature tended to increase with age in both WT and *Mtm1*^−/y^ mice treated with tamoxifen.

In striking contrast with its beneficial actions in *Mtm1*^−/y^ mice, tamoxifen caused generalized muscle hypotrophy in WT mice, which was only partly explained by decreased body weight (Supplementary Table 2, 3). In WT triceps at D42, this translated into decreased absolute force output (Fig. 2a, d), which correlated with decreased muscle mass (Fig. 2b, e). However, specific force was not altered suggesting that intrinsic mechanical properties of WT myofibers were globally preserved. The specific phasic and tetanic tensions of WT mice augmented with age independently of tamoxifen (Fig. 2c, f).

### Tamoxifen improves leg muscle structure and ultrastructure

**Fig. 3:**
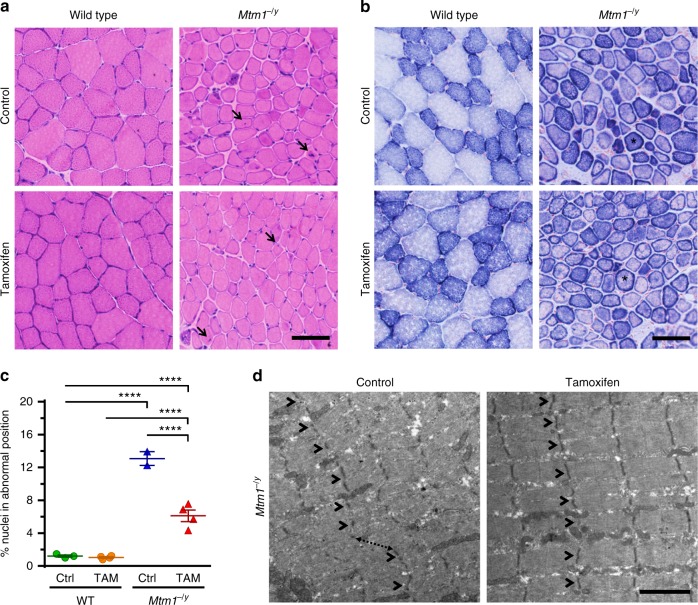
Tamoxifen mitigates muscle structure and improves sarcomeric organization in XLMTM mice. Muscle structure and sarcomere ultrastructure were examined by histology and transmission electron microscopy, respectively, in 42-day-old (D42) mice either untreated (control; Ctrl) or treated with tamoxifen (TAM; 30 mg kg^−1^ of diet). **a** Representative pictures of hematoxylin–eosin stained sections from the tibialis anterior (TA) of D42 mice. Note the small size of the *Mtm1*^−/y^ myofibres and the presence of mislocalized nuclei, a hallmark of *Mtm1*^−/y^ mice and XLMTM patients (arrows). **b** Representative pictures of succinate dehydrogenase (SDH) activity in TA sections of D42 mice, demonstrating abnormal distribution of oxidative staining. The intense staining forming a ring at the periphery of many *Mtm1*^−/y^ myofibres (examples shown by asterisks) is due to accumulated mitochondria and other organelles and is a hallmark of the pathology. **c** The percentage of nuclei abnormally positioned (either internally or centrally located) in TA myofibres of D42 mice were counted from hematoxylin–eosin-stained sections. That feature was reduced by 53.3% with TAM. Data represent the mean ± s.e.m. of *n* = 2–4 TA per group. ****P ≤ 0.0001. One-way ANOVA followed by Fisher’s LSD post-test. **d** Sarcomere ultrastructure revealed by transmission electron microscopy in the TA from untreated (control) and tamoxifen-treated *Mtm1*^−/y^ mice at D42. Note overall disorganization of the sarcomeres in untreated TA with disruption of the Z-lines (structures running perpendicular to the sarcomeres and holding myofibrils together; arrowheads) and shifted sarcomeres (dashed arrow). Tamoxifen ameliorated the organization of the sarcomeres as demonstrated by in-register Z-lines in adjacent myofibrils (arrowheads). The bar represents 50 µm in **a** and **b**, and 2 µm in **d**

Histological analyses showed that the tibialis anterior (TA) muscle of *Mtm1*^−/y^ mice contained many fibers in which organelles, including nuclei and mitochondria, were abnormally distributed, forming necklace patterns of oxidative staining essentially in large-caliber fibers^41^. Tamoxifen did not correct these specific features and had a minor impact on fiber type composition (Fig. 3a, b; Supplementary Figure 2). However, tamoxifen reduced by ~50% the number of myofibers displaying nuclei in abnormal position, a prominent histological feature of XLMTM (Fig. 3c). Tamoxifen also induced overall improvement of the myofibrillar structure, including improved organization of the Z-lines, highly ordered protein complexes that keep the sarcomeres in register, and better defined M-line, A-band, and I-band (Fig. 3d; Supplementary Figure 3).

### Tamoxifen reduces disease modifiers and alters ER levels

**Fig. 4:**
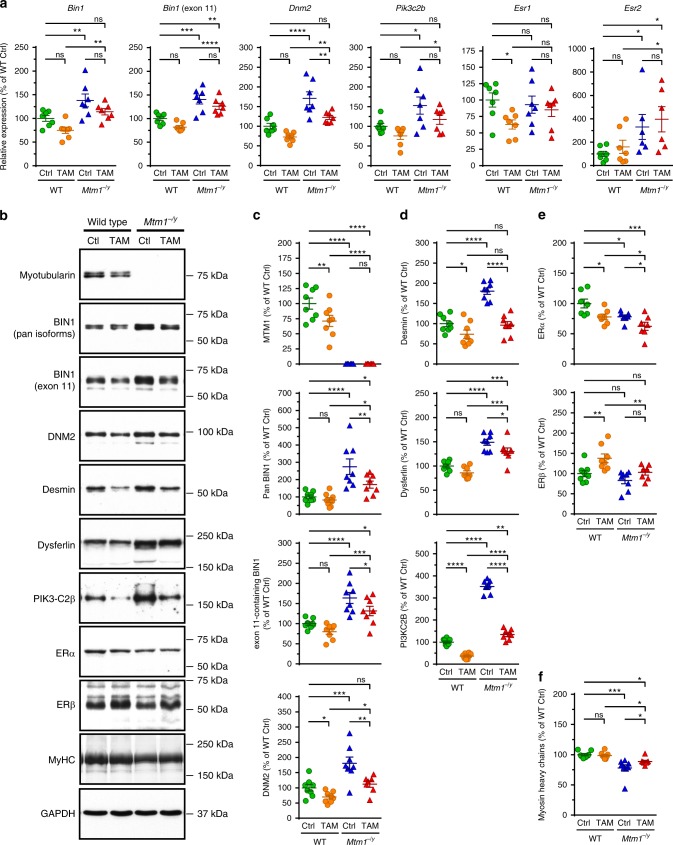
Tamoxifen reduces the expression of XLMTM disease modifiers and alters the expression of estrogen receptors. The mRNA and protein levels of known XLMTM disease modifiers and of estrogen receptors, which mediate most tamoxifen actions, were determined in gastrocnemius muscle of 42-days old (D42) wild type (WT) and *Mtm1*^−/y^ mice, either untreated (control; Ctrl) or tamoxifen (TAM)-treated (30 mg kg^−1^ of diet). **a** mRNA levels normalized to glyceraldehyde-3-phosphate dehydrogenase (*Gapdh)*. From left to right, relative expression (as percentage of WT Ctrl) of mRNA encoding amphiphysin 2/BIN1 (*Bin1*; all transcripts), muscle-specific BIN1 (*Bin1;* exon 11-containing transcript), dynamin-2 (*Dnm2*), PI3KC2B (*Pik3c2b*), estrogen receptor (ER) α (*Esr1*), and ERβ (*Esr2*). **b** Representative immunoblots of proteins of interest, as indicated. DNM2: dynamin 2; MyHC: myosin heavy chains; GAPDH: glyceraldehyde-3-phosphate dehydrogenase. Position of molecular weight markers (kDa) is shown. **c**–**f** Levels (normalized to GAPDH and expressed as percentage of WT Ctrl) of proteins selected for their role in XLMTM and tamoxifen signaling. **c** Proteins involved in the “MAD”-pathway. From top to bottom: myotubularin (MTM1), amphiphysin 2/BIN1 (pan-isoforms), amphiphysin 2/BIN1 (muscle-specific isoform), dynamin-2 (DNM2). **d** Other disease modifiers and protein deregulated in absence of MTM1. From top to bottom: desmin, dysferlin, PI3KC2B. **e** Estrogen receptors. Top: ERα; bottom: ERβ. **f** Myosin heavy chains, a major constituent of sarcomeres. Data represent the mean ± s.e.m. of *n* = 6–8 muscles per group. *P ≤ 0.05; **P ≤ 0.01; ***P ≤ 0.001; ****P ≤ 0.0001; ns non-significant. One-way ANOVA followed by Fisher’s LSD post-test

We examined the consequence of tamoxifen treatment on muscular transcript and protein levels of targets selected for their known contribution to CNM pathogenesis, for modulating *Mtm1*^−/y^ mouse phenotype, or mediating tamoxifen actions (Fig. 4; Supplementary Tables 4, 5). In the *Mtm1*^−/y^ mouse, as well in CNM patients, both BIN1 and DNM2 are overexpressed. Here we report that BIN1 mRNA is also more abundant in *Mtm1*^−/y^ mouse muscle. Overall, in both WT and *Mtm1*^−/y^ gastrocnemius muscles, tamoxifen tended to down-regulate BIN1 and DNM2 (Fig. 4a–c), both postulated to act downstream of myotubularin^42^. Of note, the ability of tamoxifen to reduce DNM2 levels was reproduced, at least partly, in a human muscle cell line established from an XLMTM individual^43,44^ (Supplementary Figure 4; see Supplementary Tables 6, 7 for details on the human muscle cell lines and the primers used for RT-PCR). In mice, tamoxifen also significantly corrected the elevated levels of desmin (a muscle-specific intermediate filament that controls nuclei positioning via binding myotubularin^45^), of PI3KC2Β (an enzyme whose genetic muscle-specific ablation rescues the disease^40^) and of dysferlin (involved in membrane repair and t-tubule biogenesis^46,47^) (Fig. 4a, b, d). Muscle extracts from untreated *Mtm1*^−/y^ mice massively accumulated a putative dysferlin degradation product (~160 kDa) whose proportion was reduced to near-normal levels with tamoxifen (Supplementary Figure 5). Tamoxifen decreased ERα but not ERβ protein levels, presumably via post-transcriptional mechanisms since these effects were not mirrored by mRNA expression (Fig. 4a, b, e). Tamoxifen also partly restored sarcomeric myosin content, which was significantly reduced in *Mtm1*^−/y^ muscle (Fig. 4b, f).

### Tamoxifen increases triad density and restores EC coupling

**Fig. 5:**
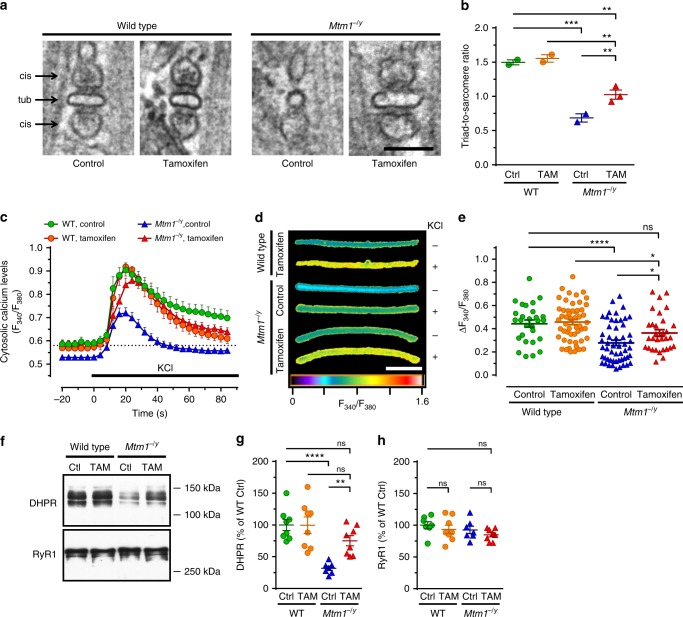
Tamoxifen improves triad density and excitation-contraction coupling, Triads and excitation–contraction coupling were examined in 42 days old (D42) mice either untreated (control; Ctrl) or treated with tamoxifen (TAM; 30 mg kg^−1^ in diet). **a** Triads—specialized membrane structures made of a t-tubule (tub) flanked by terminal cisternae (*cis*) arising from the sarcoplasmic reticulum and controlling Ca^2+^ release—were visualized by transmission electron microscopy (TEM) in the tibialis anterior (TA) of mice at D42. Note the abnormal shape of the remaining triads in the *Mtm1*^−/y^ mouse and recovery with tamoxifen treatment. The bar represents 100 nm. **b** The number of well-positioned triads per sarcomere unit was determined from TEM pictures in TA from wild type (WT) and Mtm1^−/y^ mice at D42. TAM partly rescued the much decreased triad density in *Mtm1*^−/y^ mice. Mean ± s.e.m. of *n* = 2–3 mice. **c**–**e** Excitation–contraction coupling assessed via live imaging of Ca^2+^ fluxes induced by KCl depolarization in single FDB myofibers (see Online Methods for details). **c** Average traces of the responses of FDB fibers to KCl, an experimental setting that mimics muscle depolarization (*n* = 17–29 fibers). **d** Representative images (pseudo-colored) illustrating cytosolic Ca^2+^ levels in FDB fibers at baseline (−; before KCl pulse) and at the peak of KCl-induced response (+). The bar represents 100 μm. **e** Quantification of cytosolic Ca^2+^ at the peak response. TAM enhanced *Mtm1*^−/y^ cytosolic Ca^2+^ to levels found in WT. Data shown in e represent, from left to right, the mean ± s.e.m. of *n* = 29, 53, 53, and 31 fibers. **f** Representative western blots of DHPR and RyR1. **g**, **h** Quantification of DHPR and RyR1, respectively. Mean ± s.e.m. of *n* = 7 muscles. *P ≤ 0.05; **P ≤ 0.01; ***P ≤ 0.001; ****P ≤ 0.0001; ns non-significant. One-way ANOVA followed by Fisher’s LSD post-test

Well-formed triads are required for efficient calcium release from intracellular stores and subsequent muscle contraction in response to nerve stimulation. Tamoxifen restored triad quantity and improved their morphology (Fig. 5a, b). Consistent with these findings, tamoxifen normalized the amplitude of calcium fluxes elicited by depolarization in myofibers from *Mtm1*^−/y^ mice (Fig. 5c–e). Moreover, tamoxifen almost corrected the levels of DHPR, a voltage-sensitive calcium channel that tightly regulates excitation–contraction (EC) coupling. The DHPR effector, RyR1, was found at normal levels in all groups (Fig. 5f–g).

### Long-term tamoxifen mitigates myopathic feature progression

**Fig. 6:**
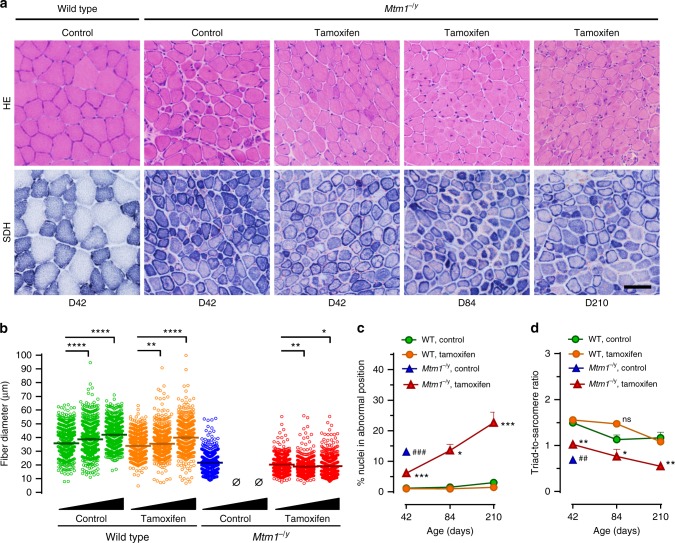
Myopathic features after long-term tamoxifen treatment, Muscle structure and triads were examined by histology and transmission electron microscopy, respectively, in wild type (WT) and *Mtm1*^−/y^ mice during long-term treatment with tamoxifen (30 mg kg^−1^ in diet). **a** Representative pictures of tibialis anterior (TA) sections from untreated WT and *Mtm1*^−/y^ mice at D42, and tamoxifen-treated *Mtm1*^−/y^ mice at D42, D84, and D210 stained with hematoxylin–eosin (HE) or for succinate dehydrogenase (SDH) activity. Muscle structure of *Mtm1*^−/y^ mice was stable over the duration of tamoxifen treatment. The bar represents 50 µm. **b** Scatter plots showing the distribution of TA myofiber diameter in mice at D42, D84, and D210 (black triangles illustrate increasing age). Fiber size remained almost constant from D42 to D210. *N* = 600 myofibers per group. **c** The percentage of abnormally positioned nuclei (either internally or centrally located) in TA myofibres of mice at D42, D84, and D210 were counted from hematoxylin–eosin-stained sections. The density of abnormally positioned nuclei increased significantly with ageing. Mean ± s.e.m. of *n* = 2–5 TA per group. **d** The number of well-positioned triads relative to sarcomere number, determined from TEM pictures in TA from wild type and *Mtm1*^−/y^ mice, showed no significant change from D42 to D84. Black triangles illustrate increasing age (42–84 and 210 days). Mean ± s.e.m. of *n* = 2–3 TA per group. *P ≤ 0.05; **P ≤ 0.01; ***P ≤ 0.001; ****P ≤ 0.0001. ^##^*P* ≤ 0.01; *Mtm1*^−/y^ control vs tamoxifen at D42. ns non-significant; Ø no surviving mice. One-way ANOVA followed by Fisher’s LSD post-test

TA muscles of *Mtm1*^−/y^ mice treated until D84 and D210 still contained small caliber myofibers with abnormally distributed organelles (Fig. 6a, b). The diameter of TA myofiber significantly increased with age in WT mice while it remained virtually stable over time in tamoxifen-treated *Mtm1*^−/y^ mice (Fig. 6c). At D42, tamoxifen more than halved myofibres with nuclei in abnormal position in *Mtm1*^−/y^ mice. Then, these pathological myofibres gradually accumulated as treated *Mtm1*^−/y^ mice became older (Fig. 6d). At D42, tamoxifen reduced by 42%, the deficit in triad number of *Mtm1*^−/y^ mice. Between D42 and D210, triad number declined similarly in both WT and *Mtm1*^−/y^-treated mice (Fig. 6e).

## Discussion

XLMTM is a rare myopathy due to the lack of the lipid phosphatase myotubularin (MTM1)^1,48^. Most affected boys die during early infancy^6^. Despite extremely high unmet medical need, very few pharmacological options have been explored so far in animal models^49,50^ or in CNM patients^51^.To date, no approved pharmacological treatment has been shown to alleviate the symptoms and increase the life expectancy of the patients.

The rationale for repurposing tamoxifen for XLMTM was based on previous success of our group with the drug in several murine models of debilitating muscular diseases, including *mdx*^*5Cv*^ mice, a model of DMD^36^. We reasoned that the many protective actions exerted by tamoxifen on diverse muscular conditions might provide a therapeutic avenue for XLMTM. Our findings largely support this view.

In brief, oral tamoxifen, administered from shortly after weaning (i.e., after *Mtm1*^−/y^ mice started to develop overt muscular symptoms), improved overall body strength, increased force generation in leg muscles, slowed disease progression through clinical stages, and importantly, prolonged the lifespan several-fold. These functional improvements correlated with reduced number of centrally located myonuclei, better excitation–contraction coupling, and normalization of molecular markers of the disease, all of which likely contribute to tamoxifen-mediated protection.

Efficient transduction of the muscular action potential into release of Ca^2+^ from sarcoplasmic reticulum stores is a critical step in excitation–contraction coupling (ECC) and a major determinant of force generation. Triads are key structures in this process. Triad disorganization and impaired ECC have a major role in the profound muscle weakness displayed by *Mtm1*^−/y^ mice and is associated with a decreased level of DHPR^52–55^. Tamoxifen not only restored about 42% of triad number deficit in the TA, but also almost fully normalized DHPR expression and dramatically enhanced Ca^2+^ release in isolated *flexor digitorum brevis* (FDB) fibers exposed to a physiological stimulus that triggers DHPR-mediated RyR activation. Altogether, beneficial actions of tamoxifen on triad count, structural components, and function very likely explain the much improved muscle function of *Mtm1*^−/y^ mice. The augmented muscle function was measured not only locally in the triceps, but also at the whole body level with the grid hanging test, suggesting that tamoxifen-mediated enhancement of ECC took place in the whole musculature. Moreover, *Mtm1*^−/y^ diaphragm weight was gradually increased over time.

Noteworthy, although body weight deficit was not corrected and improved muscle structure and muscle force did not reach WT level, the lifespan of tamoxifen-treated *Mtm1*^−/y^ mice was extended by 6.4-fold (45 days median survival of untreated KO vs 290 days) on high dose tamoxifen. Overall, it suggests that the beneficial effects of tamoxifen on life-sustaining physiological functions are sufficient to greatly extend the lifespan of the animals. Most morphological, functional, and histological outcomes were stable until around 210 days of age. Preliminary observation of long-term *Mtm1*^−/y^ mouse survivors (4 mice; average 369 days; range 271–464 days; i.e., up to 10-fold the median survival of untreated mice) suggested that the beneficial effects of tamoxifen persisted on extended periods of time.

Here we also investigated potential molecular mechanisms of the rescue. Our earlier work with tamoxifen in *mdx*^5*Cv*^ mice^36^ provided evidence that tamoxifen (i) is pro-estrogenic on striated muscles (as on bone and uterus^56^), a view supported by a recent meta-analysis in treated women^57^, and (ii) exerts beneficial actions through high affinity estrogen receptors. Typical of hormone-dependent receptor degradation, binding of tamoxifen (or its active metabolites) to ERα likely explains the lower levels of that receptor in muscles of treated *Mtm1*^−/y^ mice mice^58–60^.

Myotubularin is intricately linked to BIN1 and DNM2, which collectively control membrane dynamics from production of phosphoinositides to membrane curvature and vesicle fission^42^. Noteworthy, mutations in BIN1 and DNM2 cause autosomal forms of CNM^42^. MTM1 and BIN1 were proposed to act as negative regulators of DNM2 in muscle. Accordingly, diminishing DNM2 rescues the phenotype of *Mtm1*^−/y^ and *Bin1*^−/−^ mice^15–17^. We found that tamoxifen almost normalized BIN1 and DNM2. On their own, these molecular actions taking place just downstream of MTM1 might explain most tamoxifen-driven benefits. Furthermore, we showed that tamoxifen also reduced DNM2 levels in a human muscle cell line established from an XLMTM individual^43,44^. This further demonstrates the value of the *Mtm1*^−/y^ mouse as a model for XLMTM^15,38–40^ and supports future translation of tamoxifen efficacy findings from *Mtm1*^−/y^ mice to XLMTM patients.

Interestingly, a recent study in MCF7 cells linked DNM2 reduction to impaired autophagy—a prominent feature in *Mtm1*^−/y^ mice^39,61^—and to ERα accumulation in response to estradiol^62^. Although impaired autophagy have been reported in *Mtm1*^−/y^ mice, DNM2 levels are usually increased (and not reduced) in XLMTM and relevant model organisms. In line with these reports, our observations suggest that other mechanisms prevail in *Mtm1*^−/y^ muscle since tamoxifen (i) decreased not only DNM2 but also ERα levels, and (ii) improved overall muscle architecture and nuclei positioning, hardly consistent with further worsening of autophagic flux subsequent to DNM2 down-regulation.

Phosphatidylinositol 3-phosphate (PtdIns3*P*) is a main substrate of myotubularin and is produced by PI3-kinases including class II PI3KC2Β. Consistent with the hypothesis that altered PtdIns3*P* level account for myotubular myopathy, recent studies showed that inhibiting or down-regulating PI3KC2Β rescued the phenotype of *Mtm1*^−/y^ mice, likely via improvement of ECC^40,55^. The PI3KC2Β kinase was over-expressed several-fold in *Mtm1*^−/y^ gastrocnemius muscle. Remarkably, we found that tamoxifen almost normalized PI3KC2Β expression, thereby very likely contributing to the overall protection exerted by tamoxifen. It is not established, however, if the reduced levels of PI3KC2B is a direct effect of tamoxifen or if it is a secondary consequence of an overall improvement of muscle structure and function. Of note, our findings on PI3KC2B levels diverge from those by Dr. James Dowling and colleagues (see ref. ^63^), suggesting they might be dependent on the mouse genetic background and/or disease severity.

Desmin is a muscle-specific intermediate filament that shows discrete sarcomeric expression. It helps maintaining the myofibrils in-register and ensuring mechanical integrity of the myofibers during contraction. In *Mtm1*^−/y^ muscle and in XLMTM patients, desmin level is increased^45,64^ and its distribution resembles that in immature myotubes^44,64^. Desmin also controls nuclei and mitochondria positioning, a function that is altered by mutations in *MTM1*^45^. We showed here that tamoxifen normalized the elevated levels of desmin in *Mtm1*^−/y^ mice and this correlated with improved nuclei position and better sarcomeric structure, along with partial correction of myosin heavy chain levels, the major component of myofibrils.

Although tamoxifen significantly reduced DNM2, PI3KC2Β and desmin in muscle of both WT and *Mtm1*^−/y^ mice, these molecular changes led to dramatically different consequences. In *Mtm1*^−/y^ mice, normalization of elevated levels of these proteins likely improved the phenotype. By contrast, muscle growth and function in normal mice was impaired with tamoxifen. We speculate that this is a consequence of the reduction of these proteins and of MTM1 to sub-optimal levels. Considering the roles of the MTM1–BIN1–DNM2 pathway in t-tubule biogenesis and in excitation-contraction coupling^17,52,53,65^, it is possible that muscle cramps experienced by breast cancer patients^66–68^ result from tamoxifen-induced changes in the levels of these proteins. By contrast, we believe that tamoxifen-induced muscle atrophy observed in WT mice is likely species-specific as, to our knowledge, alteration of muscle volume has not been reported in human, not even in breast cancer patients having been on tamoxifen for several years.

In this study, we show that tamoxifen, given orally to a mouse model of XLMTM, considerably ameliorated muscle function and prolonged survival. As reported earlier in a murine model of DMD^36^, tamoxifen also caused relative hypertrophy of the diaphragm of aged *Mtm1*^−/y^ mice, which warrants further investigation for assessing the potential benefit on respiratory function. The most efficacious dose tested (30 mg of tamoxifen per kg of diet, yielding 4–6 mg of tamoxifen per kg of body weight per day) was similar to that producing optimal disease prevention in a mouse model of DMD^36^. Considering differences in drug exposure between species, this dose is clinically relevant as it matches that used for breast cancer therapy, compassionate use for DMD boys and TAMDMD, the phase 3 clinical trial (respectively ClinicalTrials.gov identifiers NCT02835079 and NCT03354039). Growth pattern in tamoxifen-treated mice was not restored to normal, which should be distinguished from a potential toxicity of tamoxifen on growth. As tamoxifen modulates molecular events downstream of myotubularin, not all pathogenic features were corrected to normal values as would be expected with gene-restoration therapies^13^. Interestingly, tamoxifen has been used as an alternative to growth hormone in short boys^31^ and improved predicted adult height in girls with McCune–Albright syndrome^35^. Thus, side effects related to suboptimal growth of XMLTM patients in future tamoxifen trials are extremely unlikely.

Interestingly, during the course of our study, Professor James Dowling and collaborators (Sick Kids Hospital, Toronto, Canada) made observations similar to ours with tamoxifen in *Mtm1*^−/y^ mice, confirming the clinically relevant findings that we present here.

Importantly, tamoxifen is a readily available EMA- and FDA-approved drug used for several decades for treating breast cancer^19–21^. It is also efficacious in other hormone-related disorders^22–27^and is safe in diverse male pediatric conditions^28–35^. We believe that the Orphan Drug Designation recently granted by the EMA for tamoxifen use in DMD^36,37^, the launching of the TAMDMD trial, and simultaneous report of the therapeutic potential of tamoxifen in *Mtm1*^−/y^ mice by two independent groups should encourage priority consideration for clinical evaluation for XLMTM. Tamoxifen fulfils all criteria^18^ for timely repurposing as a symptomatic treatment for XLMTM, either as a monotherapy or combined with AAV-*MTM1* restoration or DNM2 downregulation approaches that will hopefully prove efficacious in clinical trials. Tamoxifen might also prove useful in other myopathies where t-tubule defects secondary to increased DNM2 levels contribute to the pathogenesis, such as BIN1-related CNM and caveolin-3 and dysferlin-related myopathies^15,17,65,69^.

## Methods

### Animals and housing conditions

All procedures involving mice complied with the Swiss Federal Law on Animal Welfare. Procedures were reviewed by the veterinary office of Geneva and authorized under the license numbers GE/61/16 and GE/13/17.

*Mtm1*-deficient mice in the 129Pas genetic background were obtained from Dr. J. Laporte (IGBMC, Illkirch, France). A colony was established and maintained at the animal facility of the Geneva-Lausanne School of Pharmaceutical Sciences by breeding wild type males with *Mtm1*^+/−^ heterozygous females. The *Mtm1*^+/y^ (wild type) and *Mtm1*^−/y^ (mutant) males in the progeny were used in experiments. Mice were housed on wood granule bedding in either Eurostandard type II or type III polycarbonate cages equipped with filter tops (Indulab AG, Gams, Switzerland). They were kept on a 12-h dark/12-h light cycle, under constant temperature (22 ± 1 °C) and humidity (50–60%). Mice were allowed unlimited access to food and water throughout the study. Environment enrichment consisted in a Mouse House (Indulab AG), shredded paper, and cylinders of pure cellulose (Cocoon, Datesand, Manchester, UK).

### Genotyping

Toe biopsies were taken at 7–10 days post-natal for combined genotyping and permanent marking of individuals. DNA was prepared using DirectPCR Lysis Reagent/Mouse Tail (AxonLab AG, Le Mont-sur-Lausanne, Switzerland) according to the manufacturer’s instructions. The final lysate was diluted fivefold in molecular biology grade water. One μL of DNA was used for PCR amplification using ready to load 2x GoTaq® Master Mix (Promega, Dübendorf, Switzerland). The following primers (Microsynth AG, Balgach, Switzerland) were added: MTM1 Forward 1 (5′-AGACAGTGATGCACAGAGAGGAG-3′), binding upstream of exon 4 (used at 0.25 μM); MTM1 Forward 2 (5′-AATGGCCCCATTAAGGGAAG-3′), binding within exon 4 (used at 0.50 μM); MTM1 Reverse (5′-GTGTGCATGTTTGGACCATGG-3′), binding downstream of exon 4 (used at 0.50 μM). The PCR reaction (10 μL final volume) was run on a Biometra Tpersonal thermocycler (AxonLab) as follows: initial denaturation at 95 °C for 5 min; amplification: denaturation at 95 °C for 20 s, annealing at 55 °C for 30 s, elongation at 72 °C for 60 s, 35 cycles; final elongation at 72 °C for 5 min; holding temperature, 10 °C. PCR products were analyzed on 1% agarose gels containing SYBR Safe DNA Gel Stain (Thermo Fisher Scientific AG, Reinach, Switzerland), at a 1:20,000 dilution. The mutant allele was identified as a band of 165 bp and the wild type one as a band of 437 bp (a band of 920 bp arising from the primers binding outside exon 4 was expected but not efficiently amplified).

### Groups and treatments

*Mtm1*^+/−^ carrier females are clinically healthy. Only male mice were used in experiments. Male pups were weaned on post-natal day 23 ± 1, at which time treatments were initiated. Randomization was not feasible because small cohorts were generally produced and each group had to comprise enough wild type mice to foster the mutants. Sub-groups of mice, usually made of 2–4 wild type and 1–4 *Mtm1*^−/y^ mice, were given pellets of diet supplemented with different amounts of tamoxifen citrate (Haohua Industry Co., Jinan City, China) to achieve to 3, 10, and 30 mg of tamoxifen free base per kg of chow. The modified chow was prepared by Cargill, Provimi-Kliba AG (Kaiseraugst, Switzerland).

Specific care was systematically implemented in order to restrict suffering of the fragile *Mtm1*^−/y^ mice. This included food pellets placed inside the cages, bottles equipped with long nozzles, bedding granules to raise the ground and facilitate access to drinking water, nestling and enrichment materials, and non-experimental chaperone mice in case not enough wild type littermates were generated along with the *Mtm1*^−/y^ mice.

For survival analyses, mice were followed up to 200–210 days of age. For the other experiments, treatments were maintained until post-natal day 42 (±1) days (D42, young mice), at which time around 60% of the untreated *Mtm1*^−/y^ mice were still alive; 84 (±2) days (D84, adult mice), at which time none of the untreated *Mtm1*^−/y^ mice survived; and 210 (±3) days (D210, old mice).

### Longitudinal monitoring of disease progression

Body weight, food consumption, and clinical score were recorded three times per week on each sub-group of mice. Food consumption (in g diet per g body weight per day) was calculated from the amount of diet (in g) consumed by each cage (average total body weight in g) during periods of 2–3 consecutive days. Disease progression was assessed three times per week using a clinical grade scale adapted from that described earlier^38^. In brief, the mice were given a score of 1 (normal function of hind limbs), 2 (difficulty in spreading toes), 3 (evident weakness in legs), 4 (paralysis of one hind limb), or 5 (both hind limbs are paralyzed). Moribund mice were killed, usually soon after reaching stage 5.

Mice were subjected once a week to a horizontal grid-hanging test that measured their ability to sustain their own body weight against gravity. In brief, a mouse was placed on the center of a wire grid (dimension 35 × 50 cm, made of 1 mm-diameter metal wire and forming a 1.26 cm square mesh). The grid was gently inverted upside-down and maintained approximately 50 cm above a thick layer of soft bedding material to avoid causing damage to the animals in case of fall. The time until which the mouse fell from the grid was recorded. In the event of a fall, the timer was stopped, the grid put back to its original position, the mouse promptly returned to the grid and the test was continued. The test was ended when the final time score (set at 60 s) was reached or when the mouse was not able to hold the grid for more than 10 s between consecutive falls.

### Electrically evoked contractions of the triceps surae

Muscle mechanical properties of the triceps surae (hereafter referred to as “triceps”) were recorded in situ in deeply sedated, freely breathing mice without disturbing triceps innervation and blood supply. The triceps is a large muscle group that makes up most of the calf volume and comprises the gastrocnemius, the plantaris and the soleus muscles. Its balanced composition of slow- and fast-contracting fibers makes it similar to most human leg muscles, hence its value for physiology, experimental pharmacology and evaluating therapeutics. In an isometric setting such as the one we used, the muscle is set to a fixed length. Electrical stimulation causes attempts of the muscle to contract; the mechanical tension (“force”) exerted on its extremities is measured via a force transducer. Meaningful parameters are determined from two extreme mechanical responses that are phasic and tetanic contractions. A phasic (also known as twitch) contraction consists in an elementary muscle contraction, comprising a rising phase followed by full muscle relaxation (return to baseline), from which kinetics of contraction and relaxation can be calculated. The phasic force is measured as the amplitude of the response (see Fig. 2a, b in the main text). On the other end of the spectrum, stimulating the muscle at high frequency generates a tetanic contraction, which is the maximum force that the muscle can produce (Fig. 2d, e).

At the end of the treatment period, mice were briefly sedated by inhalation of isoflurane before i.p. injection of a mixture of urethane–diazepam–buprenorphine (1.5 g kg^−1^, 5 mg kg^−1^, and 100 μg kg^−1^, respectively), ensuring deep anesthesia and adequate analgesia for over 2 h. Mechanical responses of the right triceps to electrical stimulations were recorded isometrically using a custom-made device as previously described^36,70–74^. In brief, the knee joint was firmly immobilized, and the Achilles tendon was linked to a force transducer coupled to a LabView interface (National Instruments, Austin, TX). Two thin steel electrodes were inserted into the triceps for delivering 0.5-ms pulses of controlled intensity and frequency. After manual settings of optimal muscle length and optimal current intensity, 5–6 phasic responses to single stimuli were recorded at a sampling rate of 3 kHz to determine absolute peak twitch tension, time to peak twitch tension and time for half relaxation from peak twitch tension. Then, force–frequency curves were constructed at a sampling rate of 1 kHz: the triceps was subjected to 200-ms trains of stimuli at increasing frequencies (10 to 120 Hz by increments of 10 Hz, then 150 and 200 Hz) delivered at 30-s intervals. The maximum response was taken as the absolute tetanic tension. Absolute phasic and absolute tetanic tensions were converted into specific tensions (in mN mm^−^^2^ of muscle section) after normalization for the muscle cross-sectional area. The cross-sectional area (in mm^2^) was determined by dividing the triceps mass (in mg) by the product of the optimal muscle length (in mm) and the density of mammalian skeletal muscle (1.06 mg mm^−3^).

### Blood and tissue sampling

Immediately after isometric force recordings, mice were thoracotomized, heparin (LKT labs, Enzo Life Sciences, Lausen, Switzerland) (MW 4–6 kDa; 30 μL; 30 U mL^−1^) was injected into the heart, and the descending aorta was cut. Total blood was collected in a 1.5-mL microcentrifuge tube, and centrifuged (10 min, 4 °C, 10,000×*g*). Plasma was snap-frozen in liquid nitrogen before being stored at −80 °C. Volumes of total blood and plasma were recorded. Selected muscles were quickly dissected and weighed (Supplementary Table 2 and 3). Tibialis anterior (TA) were processed for histology and transmission electron microscopy. The gastrocnemius were saved for RT-PCR and western-blot analyses. The remaining leg muscles were collected and pooled. That muscle bulk was saved for determining levels of tamoxifen and its metabolites. In some instances, flexor digitorum brevis (FDB) muscles were used for live imaging of calcium fluxes in single intact fibers.

### Histological analysis of skeletal muscle

TA muscles were embedded in tragacanth gum (Sigma-Aldrich) (5% w/v in water), frozen in liquid nitrogen-cooled isopentane and stored at −80 °C until further processing. Air-dried 10 μm-thick transverse sections were fixed with 4% PFA and stained with hematoxylin and eosin (HE) or for succinate dehydrogenase (SDH) activity using conventional procedures^16,17^. Image acquisition was performed with a NanoZoomer 2.0-HT slide scanner equipped with the fluorescence module L11600–21 (Hamamatsu Photonics). Myofiber cross-sectional area (CSA) was analyzed using FIJI image analysis software (ImageJ 1.51s version, available at http://imagej.net/) from fluorescence pictures taken from HE-stained TA sections. CSA (μm^2^) was determined from at least 300 fibers per muscle. The percentage of myofibers with abnormally positioned nuclei (either centralized or internalized) was calculated from at least 300 fibers per TA using the cell counter plugin in FIJI analysis software. The total number of myofibers in each TA was also counted using FIJI.

### Transmission electron microscopy

The proximal half of TA muscles was cut into small pieces that were fixed with 2.5% glutaraldehyde, 2.5% paraformaldehyde and 50 mM CaCl_2_ in 0.1 M cacodylate buffer (pH 7.2). Samples were post-fixed with 2% OsO_4_, 0.8% K_3_Fe(CN)_6_ in 0.1 M cacodylate buffer (pH7.4) for 2 h a t 4 °C and incubated with 5% uranyl acetate for 2 h at 4 °C. Muscles were dehydrated in a graded series of ethanol and embedded in epon resin. Thin (70-nm) sections were stained with uranyl acetate and lead citrate and examined by transmission electron microscope. The number of triads per sarcomere (around 40 per mouse) was quantified from electron micrographs using the Metamorph 3 software^15,75^. The ratio of triads/sarcomere was calculated by dividing number of triads clearly identified and correctly localized by the total number of sarcomeres present in the image^15,76^.

### Analysis of mRNA expression by quantitative RT-PCR

The left gastrocnemius muscle (non-exposed to the isometric contraction protocol) was snap-frozen in liquid nitrogen and stored at −80 °C until processed for quantitative PCR (qPCR) essentially as described^36^. The muscles were ground to a fine powder in mortars cooled in liquid nitrogen. RNA were extracted from approximately 10 mg of muscle powder using RNeasy Fibrous Tissue mini kit (Qiagen, Hombrechtikon, Switzerland). Tissue disruption was enhanced by sonicating three times for 3 s on ice. Then 100 ng of total RNA were reverse-transcribed with Super-Script II Reverse Transcriptase (Invitrogen). The resulting cDNA was subjected to quantitative PCR (qPCR) amplification using PowerUp SYBR^TM^ Green Master mix (Applied Biosystems, Thermo Fisher Scientific) according to the manufacturer’s instructions. Briefly, 10 μL reaction mixtures were prepared containing 1 μL of cDNA (equivalent to 2.5 ng of initial RNA), forward and reverse primers (500 nM each) for *Bin1* (pan isoforms), *Bin1* (isoform 8), *Dnm2*, *Esr1*, *Esr2*, *Pik3c2b* or *Gapdh* (used as the housekeeping gene) (Supplementary Table 4), the kit Master mix and RNAse-free water. They were subjected to PCR amplification using a StepOnePlus Real-Time PCR System thermocycler (Thermo Fisher Scientific) under the following conditions: initial denaturation at 95 °C for 60 s; amplification: denaturation at 95 °C for 20 s, annealing at 55 °C for 30 s, elongation at 72 °C for 60 s, 40 cycles; final elongation at 72 °C for 5 min. The expression level of each transcript was expressed relative to that of *Gapdh* using the 2^−ΔΔCt^ method^36^.

### Semi-quantitative western-blots

Gastrocnemius muscles were pulverized on liquid nitrogen and extracts were prepared as previously described^36,73,74^. The final protein concentration was adjusted to 3 mg mL^−1^ with reducing Laemmli buffer. Muscle extracts (30 to 60 μg per lane) were resolved by SDS-PAGE, and proteins were transferred onto nitrocellulose membranes using standard procedures. Equal loading and transfer efficiency were verified with Ponceau Red staining. Membranes were blocked for 1 h in TBST (20 mM Tris-base, 150 mM NaCl, 0.05% Tween-20, pH 7.5) containing 5% non-fat dry milk and incubated overnight at 4 °C with one of the primary antibodies listed in Supplementary Table 5.

After extensive wash in TBST, the membranes were incubated with a HRP-conjugated secondary reagent (Donkey anti-Rabbit (Amersham, GE Healthcare Life Sciences, Thermo Fisher Scientific), Goat anti-mouse (Bio-Rad), or kappa light chain binding protein (Santa Cruz Biotechnologies)) as required before signal detection on X-ray Fuji films (Thermo Fisher Scientific) using an enhanced chemiluminescence kit (ECL Prime Western Blotting Detection Reagent) (Amersham). The X-ray films were scanned at high resolution (720 dpi) on an Epson Perfection V750 PRO scanner using settings for transparent documents in order to avoid signal saturation and augment the linear range of the signals. Bands were quantified using ImageJ (version 1.48; freely available at http://imagej.nih.gov/ij). Specific care allowing semi-quantitative analysis has been described in details elsewhere^36,74^. Briefly, in order to allow intra-gel and inter-gel comparison and semi-quantitative analysis of the signals, the following procedure was applied: (i) for each muscle protein to be quantified, 4 gels were run simultaneously (PerfectBlue™ Dual Gel System Twin ExW S, Peqlab) and processed in parallel, (ii) the samples (6–8 muscles per group) were loaded as quadruplets, each consisting of extracts from non-treated and TAM-treated WT and non-treated and TAM-treated *Mtm1*-null mice (iii) each quadruplet was flanked by a standard extract (Std), consisting of a mixture of all muscle extracts, (iv) the portions of the gels that contained the protein of interest were transferred onto a single nitrocellulose membrane, ensuring that all samples and flanking standard extracts were simultaneously exposed to the blocking solution, primary antibodies, secondary HRP-conjugated secondary antibody, ECL reagent, and X-ray film. Signals were normalized to GAPDH and the resulting values were normalized to that of the flanking standard samples. The standard sample being a mixture of all samples to be compared the ones with the others, this ensured that its signal has an average, non-saturating, intensity. Finally, the values were expressed as the percentage of the WT control group.

Larger views of the blots for which portions are shown in Figs. 4 and 5 are presented in Supplementary Figure 6.

### Culture of isolated flexor digitorum brevis (FDB) fibers

Hind feet were dissected and immersed in transfer buffer (Hank’s buffered salt solution supplemented with 10 mM HEPES, pH 7.4). FDB muscles were dissected under a binocular microscope, transferred to warmed (37 °C) maintenance medium (Dulbecco’s Modified Eagle’s Medium (DMEM) containing 10% fetal bovine serum and 10 μg mL^−1^ ciprofloxacin (Sigma)), and 1 mg mL^−1^ collagenase type IA (Sigma) was finally added^77^. The full process took no more than 1 h. The mixture was maintained for 60 min at 37 °C with frequent shaking in a humidified incubator gassed with air containing 5% CO_2_. Then, muscles were carefully rinsed twice in warmed maintenance medium to remove enzyme excess and individual fibers were released by triturating gently with fire-polished Pasteur pipettes of decreasing opening widths. Throughout that process, aliquots of maintenance medium containing single fibers were transferred onto clean 18 mm-diameter glass coverslips coated with 15 μg cm^−2^ Matrigel (BD biosciences) in 12-well tissue culture plates. Ca^2+^ measurement was performed the next day.

### Live imaging of cytosolic calcium in FDB fibers

Ca^2+^ levels in the cytosol of FDB fibers was monitored via fluorescence microscopy using the calcium-sensitive probe Fura-2 essentially as described previously^77^. Fura-2-AM (Molecular Probes, Invitrogen, Thermo Fisher Scientific), the acetoxymethyl ester of Fura-2, was used to facilitate loading into fibers and the subsequent de-esterification and intracellular trapping. The Fura-2-AM stock solution was prepared extemporaneously by mixing Fura-2-AM (5 mM) with an equal volume of Pluronic acid F-127 (20% w/v solution, Molecular probes). Fibers were washed twice with physiological salt solution containing Ca^2+^ (PSS^+^; composition in mM: HEPES 5, KCl 5, MgCl_2_·6H_2_O 1, NaCl 145, CaCl_2_·2H_2_O 1.7, glucose 10) before incubation in the loading solution (PSS^+^ containing 2 μL mL^−1^ of the Fura-2-AM/Pluronic acid working solution; final Fura-2 concentration 5 μM). Loading was done at 37 °C for 45 min after which the cells were washed twice with PSS^+^ and Fura-2-AM allowed to de-esterify at room temperature for another 20 min. Loading and de-esterification buffers were supplemented with 100 μM *N*-benzyl-*p*-toluenesulfonamide (BTS) (Medchem Express, Lucerna-Chem, Luzern, Switzerland) to prevent fiber contraction and detachment. All loading steps were done in the dark. Coverslips holding fibers were placed in a perfusion chamber that was continuously perfused at 1.5 mL min^−1^ using a peristaltic pump. The imaging setup consisted of a high speed monochromator (Visitron Systems, Puchheim, Germany) allowing selection of alternating excitation wavelengths and an ORCA-Flash4.0LT Digital sCMOS camera mounted on an Axiovert 200 microscope (Carl Zeiss AG, Feldbach, Switzerland) equipped with objectives for high resolution fluorescence imaging. The experiments were done ratiometrically with alternating excitation wavelengths of 340 and 380 nm and emission at wavelengths above 510 nm using a long pass filter. Images were acquired every 2 s. After recording baseline calcium, fibers were perfused with a modified PSS^+^ containing high concentration of KCl (125 mM) and reduced concentration of NaCl (10 mM). Ca^2+^ increment during depolarization was calculated as the difference between resting Ca^2+^ levels and the maximum levels of Ca^2+^ reached during depolarization. Data acquisition and analysis was performed using VisiView software version 3.3.0.3 (Visitron Systems).

### Data analysis and statistics

Survival curves were analyzed using the Log-rank (Mantel–Cox) test. All other comparisons were assessed by ANOVA followed by Fisher’s least significance difference (LSD) test. The overall changes in body weights, grid test scores and clinical grades over time were assessed from the areas under the curves calculated from every mouse.

## Electronic supplementary material

Supplementary Information

Peer Review File

Supplementary Movie 1

Supplementary Movie 2

Supplementary Movie 3

Supplementary Movie 4

Description of Additional Supplementary Files

## Data Availability

The authors declare that all data supporting the findings of this study are available within the paper and its supplementary information files. A reporting summary for this Article is available as a Supplementary Information file.
