# Supplementary material for: Tamoxifen prolongs survival and alleviates symptoms in mice with fatal X-linked myotubular myopathy
Source: Nat Commun. 2018 Nov 19;9:4848. doi: 10.1038/s41467-018-07058-4 (PMC6243013; doi:10.1038/s41467-018-07058-4)
Supplement: Supplementary file 1 — Supplementary Information [file 41467_2018_7058_MOESM1_ESM.pdf]

Supplementary Information to

Tamoxifen prolongs survival and alleviates symptoms in mice with fatal X-linked myotubular myopathy

by Gayi *et al*.

Supplementary figures

Supplementary Figure 1

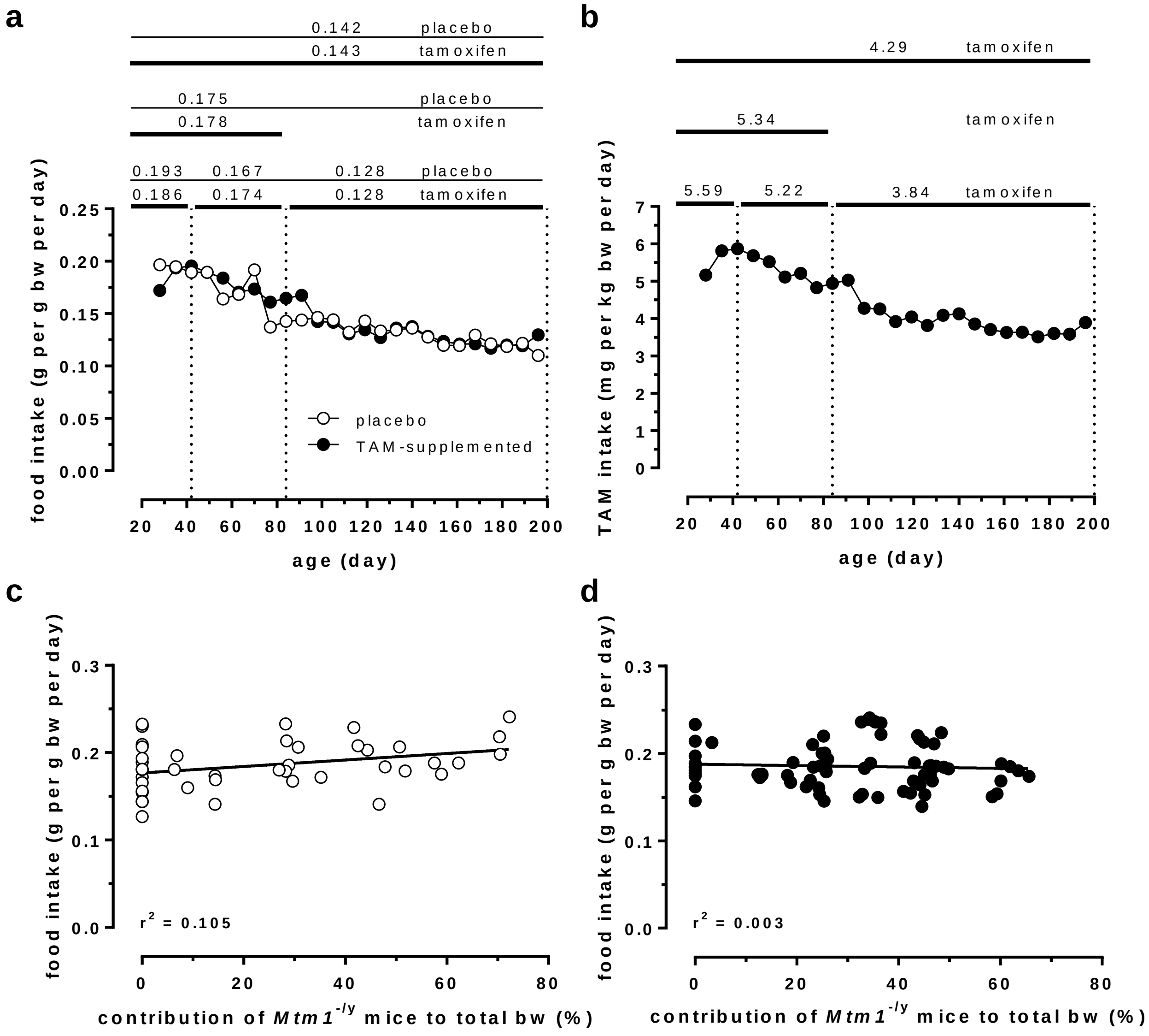

Diet and drug consumption by wild type and mutant mice. Subgroups of 2-4 wild type (WT) and 1-4 *Mtm*$^{-/y}$ mice were given pellets of placebo diet (control) or pellets of a diet supplemented with tamoxifen citrate (equivalent to 30 mg tamoxifen per kg of chow). Body weights (bw) and diet consumed were recorded 3 times a week**.** In **(a, b)**, the ages at which analyses were performed are highlighted by dotted vertical lines. The data are the average of n = 10 (control) or 14 (tamoxifen) subgroups of mice. The values above the graphs show food **(a)** or tamoxifen **(b)** intake during the time windows indicated by thick lines. **(a)** Food intake was calculated as the amount of diet consumed (in g) per gram of mouse per day. Food intake was similar on control and tamoxifen diets throughout the study. Intake was highest in young animals (approx. 0.19 $g.g^{-1}.d^{-1}$ from weaning to D42) and then decreased with age in both treatment groups (average food intake 0.128 $g.g^{-1}.d^{-1}$ in mice aged 84 to 200 days). **(b)** Tamoxifen intake (mg tamoxifen per kg bw per day) was calculated

from food intake of the treated subgroups. It slowly declined from approximately 5.6 $mg.g^{-1}.d^{-1}$ in young treated mice to 5.2 $mg.g^{-1}.d^{-1}$ in mice aged from D42 to D84, to 3.8 $mg.g^{-1}.d^{-1}$ in mice aged D84 to D200. Mice treated up to D200 were exposed to an average tamoxifen dose of 4.3 $g.g^{-1}.d^{-1}$. We wondered if drug intake was similar in WT and in $Mtm^{-/y}$ mice. We thus analyzed food intake between D23 and D84 in control **(c)** and tamoxifen-treated **(d)** mice as a function of subgroup composition. The contribution of the $Mtm^{-/y}$ mice to the total bw of the subgroups ranged from 72% to 0% (when all the $Mtm^{-/y}$ mice were dead or used in experiments and only WT mice remained). There was no correlation between food intake and the contribution of $Mtm^{-/y}$ mice to the subgroups. We thus concluded that WT and in $Mtm^{-/y}$ mice ate similar amounts of diet relative to their body weight and were thus exposed to the same amount of tamoxifen.

Supplementary Figure 2

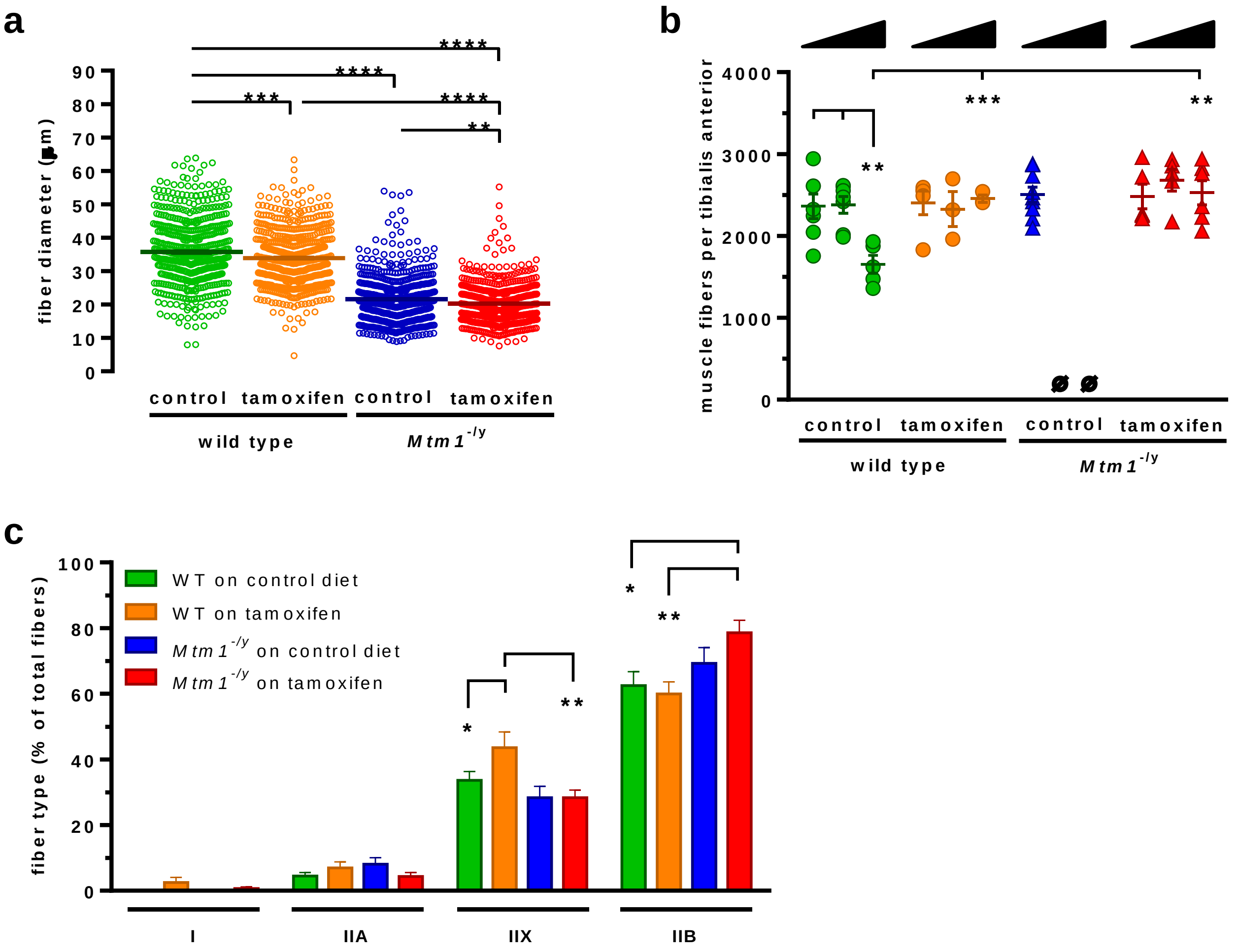

Effects of tamoxifen on morphometry and fiber type composition of murine tibialis anterior. Wild type (WT) and *Mtm$^{-/y}$* mice were fed either a control diet or a tamoxifen-supplemented diet (30 mg per kg of diet) from weaning (at D23) onward. **(a)** Fiber size (minimum Ferret diameter) in the tibialis anterior (TA) muscle was measured at D42. Data represent the individual diameter of 600 fibers (150 or 200 fibers from 3-4 TA) per group. Fibers in the TA of *Mtm$^{-/y}$* mice were significantly smaller than in WT mice. Tamoxifen slightly but significantly reduced fiber size in the TA of both WT and *Mtm$^{-/y}$* mice. **(b)** The number of myofibers in the TA muscle was counted at D42, D84 and D210 from H&E-stained sections. The number of fibers was similar between all 4 groups at D42 and between all 3 remaining groups at D84. At D210, the TA of WT mice fed the control diet showed a significant reduction in fiber count, which was prevented by tamoxifen. The number of fibers in the TA of tamoxifen-treated *Mtm$^{-/y}$* mice showed no drop over time and remained similar to that of young/adult normal mice throughout the study. **(c)** Fiber typing at D42 was performed using antibodies specific to myosin heavy chain types. In the TA of WT mice, tamoxifen caused a fast-to-slow shift: type I, IIA and IIX fibers accumulated at the expense of IIB fibers. By contrast, tamoxifen tended to cause an accumulation of type IIB fibers in the TA of *Mtm$^{-/y}$* mice with concomitant decrease of type IIX and IIA fibers. Data represent the mean ± s.e.m. of n = 3-9 muscles per group. The black triangles illustrate increasing age (42 to 84 and >200 days). **(a, b)** ** $P \leq 0.01$; *** $P \leq 0.001$; **** $P \leq 0.0001$; ns: non-significant; Ø: no surviving mice.

Supplementary Figure 3

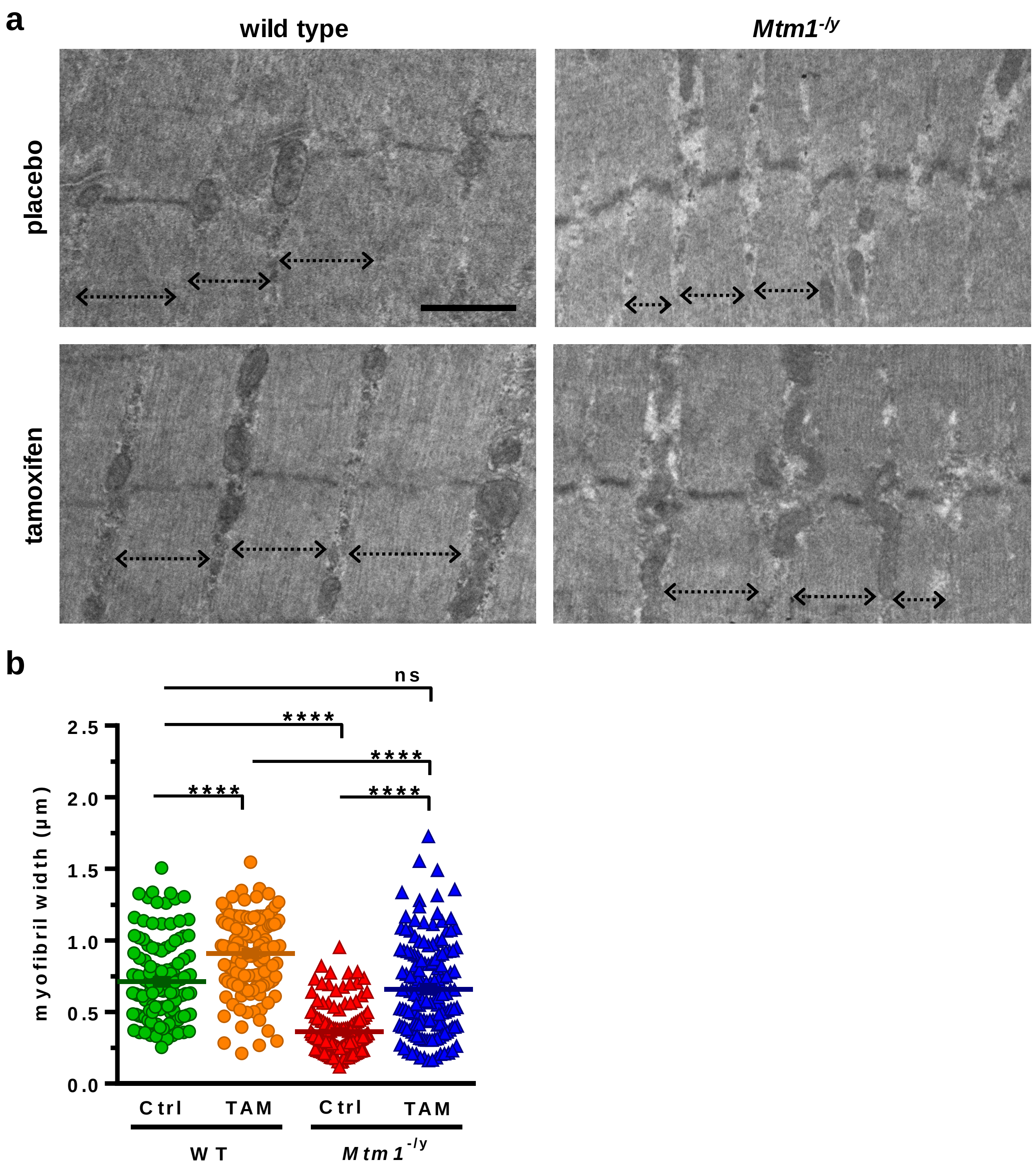

Tamoxifen increases myofibril width in wild type and XLMTM mice. Wild type (WT) and *Mtm*$^{-/y}$ mice were fed either a control diet or a tamoxifen-supplemented diet (30 mg per kg of diet) from weaning (at D23) onward. Muscle fiber ultrastructure was explored by electron microscopy in the tibialis anterior (TA) muscle at D42. **(a)** Untreated *Mtm*$^{-/y}$ mice showed disorganized sarcomeres with fuzzy Z-lines and poorly delineated M-lines, A-bands and I-bands. Tamoxifen improved the overall organization of the sarcomeres: note the better alignment of the Z-lines as well as the better definition of the M-line as reported in the main text. Tamoxifen seemed to increase the density of the

mitochondrial network in the TA of both WT and *Mtm*$^{-/y}$ mice. In untreated *Mtm*$^{-/y}$ mice, most mitochondria appeared short, rounded or enlarged, while with tamoxifen, they seemed both better shaped and better oriented along the sarcomeres. The bar represents 1 µm. **(b)** Myofibril width (dotted arrows) was measured at D42 from electron microscopy pictures similar to those shown in (a). Myofibril width was much reduced in *Mtm*$^{-/y}$ mice compared to WT mice. Tamoxifen increased myofibril width in both WT and *Mtm*$^{-/y}$ mice. Myofibril width in tamoxifen-treated *Mtm*$^{-/y}$ mice was similar to that in WT mice. Data represent the mean ± s.e.m. of n = 127-150 measurements from 2-4 muscles per group. **** $P \le 0.0001$; ns: non-significant.

Supplementary Figure 4

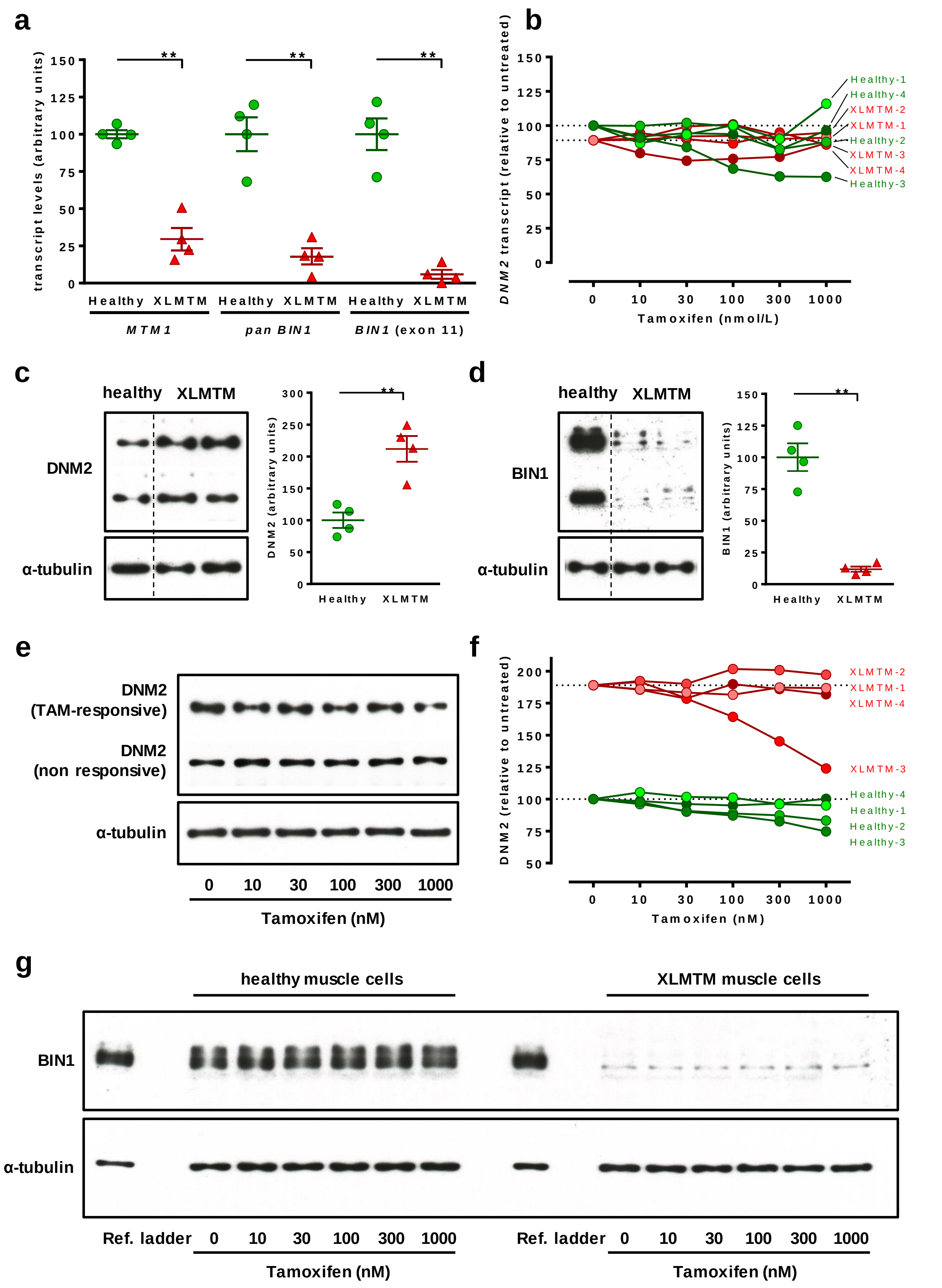

Human muscle cells recapitulate the molecular signature of tamoxifen on DNM-2 expression. Human muscle cell lines established from 4 healthy donors and 4 XLMTM individuals were maintained for 7 days in low-mitogen medium for inducing myogenic differentiation. In some experiments, the cells were exposed to tamoxifen for 5 days at the indicated concentration before extracts were prepared for quantitative RT-PCR and western-blotting analysis. Transcript levels of *MTM1*, pan *BIN1* and exon11-containing (muscle-specific) *BIN1* were all markedly reduced in untreated XLMTM lines **(a)** and their expression was not altered with tamoxifen (not shown). **(b)** On average, *DNM2* transcripts in XLMTM lines were 89% of the levels in healthy lines and were not changed by tamoxifen treatment for 5 days. **(c)** Protein levels of DNM2 in XLMTM muscle cells were almost doubled, a feature shared with the XLMTM mouse model. **(d)** Amounts of BIN1 protein in XLMTM muscle cells were strongly diminished compared to healthy muscle cells. **(c, d)** left panel: representative blots; right panel: densitometric signal quantification. Healthy and XLMTM muscle cell lines were screened for changes in DNM2 expression following exposure to tamoxifen **(e**: representative western blots; **f**: signal quantification**)**. On average, the expression of DNM2 in XLMTM lines was 189% of the levels in healthy lines **(f)**. After being exposed to tamoxifen for 5 days, healthy muscle cells showed a slight decrease in DNM2 expression (-12% on average). Out of 4 XLMTM muscle lines, 3 did not respond to tamoxifen, presumably due to phenotypic drift upon *in vitro* passaging. One XLMTM muscle cell line (XLMTM-3) showed a significant decrease in DNM2 (-35%) to levels close to those found in healthy lines. **(g)** Tamoxifen did not modify BIN1 protein levels. A reference sample (Ref.) was loaded on all gels for comparison purpose. Data are average values from 2 to 3 independent determination per cell line. ** $P \leq 0.01$; two-tailed unpaired *t*-test.

Supplementary Figure 5

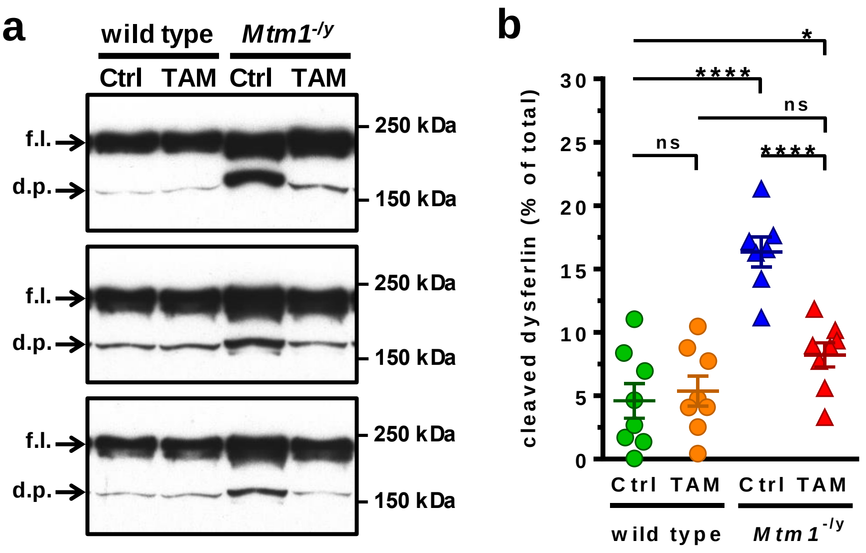

Tamoxifen corrects the abnormal pattern of dysferlin cleavage products. Wild type (WT) and *Mtm*$^{-/y}$ mice were fed either a control diet or a tamoxifen-supplemented diet (30 mg per kg of diet) from weaning (at D23) onward. Gastrocnemius muscle extracts were prepared at D42 and dysferlin expression analyzed by western blotting. **(a)** Dysferlin expression in 3 independent sets of 4 samples. Molecular weights are shown on the right-hand side. The signals corresponding to the full-length (f.l.) dysferlin are saturating as the blots were over-exposed in order to detect the putative dysferlin degradation product (d.p.), which appears as the band of approximately 160 kDa. **(b)** The putative dysferlin degradation product was quantified by densitometry and expressed as a percentage of total dysferlin. Cleaved dysferlin represented less than 5% of total dysferlin in untreated WT muscles and this was not affected by tamoxifen. Dysferlin cleavage was much higher in *Mtm*$^{-/y}$ mice, reaching 16% of total dysferlin. This excessive cleavage was prevented by 50% with tamoxifen. Data show individual values and the mean ± s.e.m. of n = 8 samples per group. * $P \leq 0.05$; **** $P \leq 0.0001$; ns: non-significant.

Supplementary Figure 6

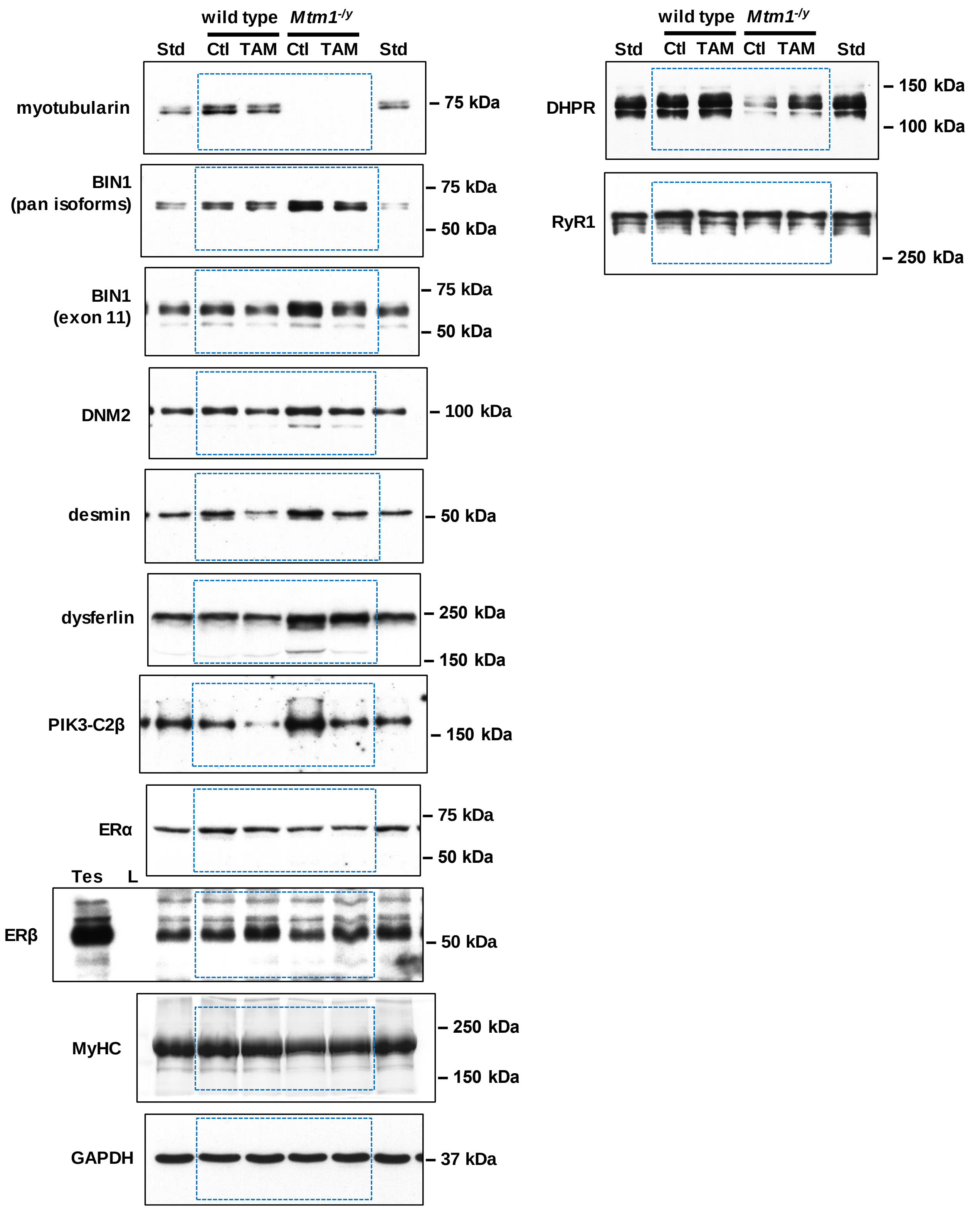

Larger views of the western blots and gels shown in the work. As described in the main text, specific care was taken in order to allow intra-gel and inter-gel comparison and semi-quantitative analysis of the western-blot signals: (i) all the samples to be compared the ones with the others were run in parallel on large gels by SDS-PAGE; (ii) the samples (6-8 muscles per group) were loaded as

quadruplets, each consisting of extracts from 1 non-treated wild type (WT) mouse, 1 TAM-treated mouse, 1 non-treated TAM-treated *Mtm1*-null (*Mtm1*$^{-/y}$) mouse and 1 TAM-treated *Mtm1*$^{-/y}$ mouse; (iii) each quadruplet was flanked by a standard extract (Std), consisting of a mixture of all muscle extracts, to which each muscle extract was normalized; (iv) at the end of the electrophoresis, horizontal gel strips containing the protein of interest were cut out and transferred onto a single nitrocellulose membrane. In order to accommodate 3-4 of such strips on a same membrane, each strip had to measure no more than 2.5 to 3 cm in height (i.e. around one-third of the whole height of the gel). In order to spare the samples, 2 or 3 proteins with different molecular weights were routinely detected from different gel strips (corresponding to high, medium, and low molecular weight proteins). Proteins of interest were detected by enhanced chemiluminescence using X-ray films. Each quadruplet flanked by Std samples was then scanned before quantification of the signals by densitometry. The portions of the representative blots that are shown in Figures 4 and 5 of the main document are delineated by blue dashed boxes in (a) and (b), respectively. All proteins were quantified by western blots, except myosin heavy chains (MyHC), which were measured from Coomassie-stained gels. In some instances, extracts from selected tissues used as positive controls were loaded on the extremities of the gels in order to ascertain the identity of the protein of interest. For example, a murine testes extract (Tes) was used as a positive control for ERβ. L: molecular weight ladder.

Supplementary Tables

Supplementary Table 1

Levels of tamoxifen and tamoxifen metabolites in plasma and in muscle tissue

	plasma levels (ng.mL^{-1})					
	wild type			*Mtm1$^{-/y}$*		
	D42	D84	D210	D42	D84	D210
**(Z)-TAM**	0.36	0.21	0.17	0.27	0.23	0.15
**(E)-TAM**	n/d	n/d	n/d	n/d	n/d	n/d
**(Z)-OHT**	0.13	0.09	0.10	0.11	0.06	0.06
**(E)-OHT**	n/d	n/d	n/d	n/d	n/d	n/d
**(Z)-NDT**	0.16	0.14	0.07	0.09	0.03	0.05
**(E)-NDT**	n/d	n/d	n/d	n/d	n/d	n/d
**(Z)-ENDO**	0.09	0.04	0.07	0.11	0.02	0.02
**(E)-ENDO**	0.06	0.01	0.03	0.07	0.01	0.00
**TOTAL**	0.80	0.49	0.44	0.65	0.35	0.28

	muscle levels (ng.g^{-1})					
	wild type			*Mtm1$^{-/y}$*		
	D42	D84	D210	D42	D84	D210
**(Z)-TAM**	9.20	7.29	6.98	7.44	6.97	6.93
**(E)-TAM**	n/d	n/d	n/d	n/d	n/d	n/d
**(Z)-OHT**	3.43	2.33	4.01	2.59	1.72	1.11
**(E)-OHT**	n/d	n/d	n/d	n/d	n/d	n/d
**(Z)-NDT**	1.22	1.00	1.04	1.38	1.24	1.18
**(E)-NDT**	n/d	n/d	n/d	n/d	n/d	n/d
**(Z)-ENDO**	4.87	2.99	2.51	2.66	0.81	0.51
**(E)-ENDO**	2.86	2.95	2.64	1.69	n/d	n/d
**TOTAL**	21.58	16.56	17.18	15.76	10.74	9.73

The table shows the levels of both the Z and E stereoisomers of tamoxifen (TAM) and three of its major metabolites (4-hydroxytamoxifen (OHT), N-desmethyltamoxifen (NDT), and endoxifen (ENDO)) measured in the plasma and in skeletal muscles (pool of leg muscles) of mice treated with (Z)-tamoxifen (30 mg per kg of diet). Values represent the average of 3 to 7 determinations. The levels of tamoxifen and of its metabolites were 24 to 39-times higher in skeletal muscle than in plasma. TAM and OHT were the major species found in both plasma and skeletal muscle. Maybe of pathophysiological relevance, TAM accounted for 42-71% of total tamoxifen species in *Mtm1$^{-/y}$* mice *vs.* only 39-45% in wild type mice, suggesting that *Mtm1$^{-/y}$* mice do not metabolize TAM as well as wild type mice do. n/d: not detected.

Supplementary Table 2

Absolute weights of mice and selected muscles

	wild type control diet	wild type tamoxifen diet	*Mtm1*$^{-/y}$ control diet	*Mtm1*$^{-/y}$ tamoxifen diet
**D42**	mean ± s.e.m.	mean ± s.e.m.	mean ± s.e.m.	mean ± s.e.m.
body weight	23.1 ± 0.5	17.2 ± 0.9 ****	14.6 ± 0.8 ****	16.6 ± 0.4 **** #
heart	110.9 ± 2.9	85.8 ± 7.5 ***	82.3 ± 3.9 ****	86.8 ± 3.3 **
diaphragm	46.8 ± 1.2	37.7 ± 1.9 ***	23.6 ± 1.3 ****	24.5 ± 1.0 ****
gastrocnemius	90.3 ± 1.8	57.0 ± 2.8 ****	29.6 ± 2.0 ****	39.5 ± 1.9 **** ##
tibialis anterior	30.9 ± 0.5	21.6 ± 1.1 ****	10.5 ± 0.5 ****	10.9 ± 0.4 ****
EDL	7.3 ± 0.2	5.4 ± 0.3 ****	3.3 ± 0.2 ****	3.8 ± 0.2 ****
soleus	4.6 ± 0.1	3.2 ± 0.2 ***	3.0 ± 0.1 ***	3.4 ± 0.1 ***
tibialis posterior	23.1 ± 0.5	16.8 ± 0.6 ****	14.5 ± 0.8 ****	16.4 ± 0.5 **** #
plantaris	12.8 ± 0.3	8.5 ± 0.4 ****	7.8 ± 0.3 ****	9.1 ± 0.2 **** ##
quadriceps	129.3 ± 3.4	84.3 ± 4.5 ****	50.2 ± 2.6 ****	62.6 ± 1.8 **** ##
triceps	111.1 ± 2.3	70.3 ± 3.4 ****	42.1 ± 2.5 ****	54.0 ± 2.2 **** ##
leg muscles	758.3 ± 18.0	506.3 ± 26.1****	271.7 ± 14.3****	349.7 ± 10.1**** ##
**D84**	mean ± s.e.m.	mean ± s.e.m.	mean ± s.e.m.	mean ± s.e.m.
body weight	25.5 ± 0.6	23.0 ± 0.9 ** ‡‡‡	n/a	14.3 ± 0.3 **** ‡
heart	116.9 ± 4.9	103.5 ± 4.9 * ‡	n/a	72.9 ± 2.3 **** ‡
diaphragm	54.0 ± 1.2	50.1 ± 2.4 ‡‡‡	n/a	25.5 ± 0.9 ****
gastrocnemius	104.7 ± 2.7 ‡‡	82.6 ± 3.6 **** ‡‡‡‡	n/a	42.7 ± 1.7 ****
tibialis anterior	37.6 ± 0.7 ‡‡‡‡	31.2 ± 1.2 **** ‡‡‡‡	n/a	11.0 ± 0.2 ****
EDL	8.7 ± 0.2 ‡‡‡‡	7.3 ± 0.2 **** ‡‡‡‡	n/a	3.5 ± 0.1 ****
soleus	5.2 ± 0.1 ‡‡	4.6 ± 0.2 ** ‡‡‡‡	n/a	2.8 ± 0.1 ****
tibialis posterior	28.2 ± 0.6 ‡‡‡‡	22.5 ± 0.9 **** ‡‡‡‡	n/a	13.4 ± 0.7 **** ‡‡
plantaris	15.9 ± 0.5 ‡‡‡‡	12.2 ± 0.4 **** ‡‡‡‡	n/a	9.0 ± 0.4 ****
quadriceps	162.3 ± 4.2 ‡‡‡‡	128.7 ± 5.1 **** ‡‡‡‡	n/a	51.1 ± 1.6 **** ‡‡‡
triceps	126.6 ± 3.6 ‡‡‡	101.1 ± 4.0 **** ‡‡‡‡	n/a	56.2 ± 1.7 ****
leg muscles	951.5 ± 20.5 ‡‡‡‡	734.1 ± 41.8**** ‡	n/a	316.1 ± 9.8 ****
**D210**	mean ± s.e.m.	mean ± s.e.m.	mean ± s.e.m.	mean ± s.e.m.
body weight	35.0 ± 2.0 ‡‡‡‡	29.1 ± 0.9 ** ‡‡‡‡	n/a	15.0 ± 1.0 ****
heart	157.6 ± 7.1 ‡‡‡‡	113.0 ± 2.8 **** ‡‡‡	n/a	88.9 ± 5.9 ****
diaphragm	54.0 ± 5.1	48.5 ± 1.7 ‡‡‡	n/a	49.1 ± 4.1
gastrocnemius	107.1 ± 4.6 ‡‡‡	73.5 ± 1.7 **** ‡‡‡‡	n/a	44.3 ± 3.9 ****
tibialis anterior	40.3 ± 1.0 ‡‡‡‡	30.7 ± 0.6 **** ‡‡‡‡	n/a	12.2 ± 0.8 ****
EDL	8.9 ± 0.2 ‡‡‡‡	7.2 ± 0.2 **** ‡‡‡‡	n/a	3.9 ± 0.2 ****
soleus	5.0 ± 0.2	4.1 ± 0.1 *** ‡‡‡	n/a	3.3 ± 0.4 ****
tibialis posterior	27.9 ± 1.0 ‡‡‡‡	19.9 ± 0.6 **** ‡‡	n/a	13.4 ± 0.7 **** ‡‡
plantaris	15.6 ± 0.4 ‡‡‡‡	11.4 ± 0.2 **** ‡‡‡‡	n/a	7.3 ± 0.5 **** ‡‡
quadriceps	167.7 ± 5.4 ‡‡‡‡	124.2 ± 2.7 **** ‡‡‡‡	n/a	50.1 ± 2.8 **** ‡‡‡
triceps	126.0 ± 3.1 ‡‡	92.7 ± 2.0 **** ‡‡‡‡	n/a	55.7 ± 4.4 ****
leg muscles	975.2 ± 27.0 ‡‡‡‡	710.7 ± 15.8**** ‡‡‡‡	n/a	318.8 ± 20.7****

The table shows mouse body weights in grams (g) and muscle weights in milligrams (mg). Data represent the mean ± s.e.m. of 6 to 28 muscles, obtained from 6 to 14 mice fed a control diet or a diet supplemented with the high dose of tamoxifen (30 mg per kg of diet). One-way ANOVA with Fisher's least significance difference (LSD) post-test. n/a: not available (no untreated *Mtm1*$^{-/y}$ mouse survived to that age). * $P \leq 0.05$; ** $P \leq 0.01$; *** $P \leq 0.001$; **** $P \leq 0.0001$; different from wild type control at any given age. # $P \leq 0.05$; ## $P \leq 0.01$; ### $P \leq 0.001$; #### $P \leq 0.0001$; *Mtm1*$^{-/y}$ control vs. *Mtm1*$^{-/y}$ tamoxifen at D42. ‡ $P \leq 0.05$; ‡‡ $P \leq 0.01$; ‡‡‡ $P \leq 0.001$; ‡‡‡‡ $P \leq 0.0001$; different from D42 (age effect for a given muscle and a given treatment group).

Supplementary Table 3

Weight of selected muscles normalized to body weight

	wild type control diet	wild type tamoxifen diet	*Mtm1*$^{-/y}$ control diet	*Mtm1*$^{-/y}$ tamoxifen diet
**D42**	mean ± s.e.m.	mean ± s.e.m.	mean ± s.e.m.	mean ± s.e.m.
heart	4.80 ± 0.08	4.96 ± 0.18	5.75 ± 0.13****	5.23 ± 0.08* #
diaphragm	2.03 ± 0.04	2.21 ± 0.12	1.63 ± 0.04****	1.47 ± 0.03****
gastrocnemius	3.91 ± 0.05	3.30 ± 0.07****	1.99 ± 0.08****	2.34 ± 0.10**** ###
tibialis anterior	1.34 ± 0.01	1.25 ± 0.04**	0.72 ± 0.01****	0.65 ± 0.02**** #
EDL	0.32 ± 0.01	0.31 ± 0.01	0.23 ± 0.01****	0.23 ± 0.01****
soleus	0.20 ± 0.01	0.19 ± 0.01	0.21 ± 0.01	0.21 ± 0.01
tibialis posterior	1.00 ± 0.02	0.98 ± 0.03	1.00 ± 0.04	0.98 ± 0.03
plantaris	0.55 ± 0.01	0.49 ± 0.01***	0.54 ± 0.01	0.54 ± 0.01
quadriceps	5.60 ± 0.10	4.87 ± 0.11****	3.39 ± 0.06****	3.72 ± 0.07**** ##
triceps	4.82 ± 0.08	4.07 ± 0.09****	2.85 ± 0.08****	3.21 ± 0.11**** ##
leg muscles	32.81 ± 0.38	29.30 ± 0.64****	18.41 ± 0.37****	20.80 ± 0.38**** ###
**D84**	mean ± s.e.m.	mean ± s.e.m.	mean ± s.e.m.	mean ± s.e.m.
heart	4.66 ± 0.19	4.50 ± 0.08‡	n/a	5.11 ± 0.08*
diaphragm	2.13 ± 0.05	2.18 ± 0.05	n/a	1.79 ± 0.05**** ‡‡‡
gastrocnemius	4.14 ± 0.10	3.58 ± 0.08*** ‡‡	n/a	3.01 ± 0.12**** ‡
tibialis anterior	1.49 ± 0.03 ‡‡‡	1.35 ± 0.03*** ‡	n/a	0.78 ± 0.01**** ‡‡
EDL	0.34 ± 0.01 ‡‡	0.32 ± 0.01*	n/a	0.25 ± 0.01****
soleus	0.21 ± 0.01	0.20 ± 0.01	n/a	0.19 ± 0.01
tibialis posterior	1.12 ± 0.03 ‡‡‡	0.98 ± 0.03**	n/a	0.94 ± 0.05****
plantaris	0.63 ± 0.02 ‡‡‡	0.53 ± 0.01** ‡	n/a	0.64 ± 0.03
quadriceps	6.42 ± 0.18 ‡‡‡‡	5.58 ± 0.10**** ‡‡‡‡	n/a	3.58 ± 0.09****
triceps	5.00 ± 0.13	4.38 ± 0.08*** ‡	n/a	3.96 ± 0.13**** ‡
leg muscles	37.63 ± 0.84 ‡‡‡‡	31.70 ± 1.32****	n/a	22.18 ± 0.61****
**D210**	mean ± s.e.m.	mean ± s.e.m.	mean ± s.e.m.	mean ± s.e.m.
heart	4.61 ± 0.14	3.90 ± 0.07**** ‡‡‡‡	n/a	5.93 ± 0.16**** ‡‡‡
diaphragm	1.60 ± 0.15 ‡‡	1.67 ± 0.04 ‡‡‡‡	n/a	3.30 ± 0.22**** ‡‡‡‡
gastrocnemius	3.10 ± 0.15 ‡‡‡‡	2.53 ± 0.05**** ‡‡‡‡	n/a	2.93 ± 0.2 ‡
tibialis anterior	1.16 ± 0.03 ‡‡‡‡	1.06 ± 0.02** ‡‡‡‡	n/a	0.81 ± 0.03**** ‡‡‡‡
EDL	0.26 ± 0.01 ‡‡‡‡	0.25 ± 0.01 ‡‡‡‡	n/a	0.26 ± 0.01 ‡
soleus	0.14 ± 0.01 ‡‡‡‡	0.14 ± 0.01 ‡‡‡‡	n/a	0.22 ± 0.02****
tibialis posterior	0.81 ± 0.03 ‡‡‡‡	0.69 ± 0.02** ‡‡‡‡	n/a	0.89 ± 0.03* ‡
plantaris	0.45 ± 0.01 ‡‡‡‡	0.40 ± 0.01** ‡‡‡‡	n/a	0.49 ± 0.02
quadriceps	4.83 ± 0.13 ‡‡‡	4.29 ± 0.09*** ‡‡‡	n/a	3.34 ± 0.12**** ‡‡
triceps	3.64 ± 0.09 ‡‡‡‡	3.20 ± 0.06*** ‡‡‡‡	n/a	3.69 ± 0.21
leg muscles	28.17 ± 0.79 ‡‡‡‡	24.51 ± 0.44**** ‡‡‡‡	n/a	21.20 ± 0.9 ****

The table shows the weight of muscles normalized to mouse body weight (mg.g^{-1}). Data represent the mean ± s.e.m. of 6 to 28 muscles, obtained from 6 to 14 mice fed a control diet or a diet supplemented with the high dose of tamoxifen (30 mg per kg of diet). One-way ANOVA with Fisher's least significance difference (LSD) post-test. n/a: not available (no untreated *Mtm1*$^{-/y}$ mouse survive to that age). * $P \leq 0.05$; ** $P \leq 0.01$; *** $P \leq 0.001$; **** $P \leq 0.0001$; different from wild type control at any given age. # $P \leq 0.05$; ## $P \leq 0.01$; ### $P \leq 0.001$; *Mtm1*$^{-/y}$ control vs. *Mtm1*$^{-/y}$ tamoxifen at D42. ‡ $P \leq 0.05$; ‡‡ $P \leq 0.01$; ‡‡‡ $P \leq 0.001$; ‡‡‡‡ $P \leq 0.0001$; different from D42 (age effect for a given muscle and a given treatment group).

Supplementary Table 4

Primers used for quantitative analysis of murine transcripts by RT-PCR

primer	exon	sequence	amplicon size (bp)
Bin1 pan-forward	2	5’-ACGAAGGACGAGCAGTTTGA-3’	101
Bin1 pan-reverse	3	5’-CAGAAGCCAGATAGGTCCGAA-3’	
*Bin1*exon 11-forward	10	5’- CTGGTCAGCCTAGAGAAGCAG -3’	88
*Bin1*exon 11-reverse	11	5’- GCAGCCGTGAGAACAGTTT -3’	
Dnm2-forward	3	5’-GCAGGAGATCGAAGCAGAGAC-3’	117
Dnm2-reverse	4	5’-GGTCGATGAGGGTCAAGTTCAA-3’	
Esr1-forward	5	5’-TGCGCAAGTGTTACGAAGTG-3’	109
Esr1-reverse	6	5’-TTTCGGCCTTCCAAGTCATC-3’	
Esr2-forward	2	5’-TCGCTTCTCTATGCAGAACC-3’	138
Esr2-reverse	3	5’-AGAAGTGAGCATCCCTCTTG-3’	
Gapdh-forward	6	5’-GTTGTCTCCTGCGACTTCA 3’	184
Gapdh-reverse	7	5’-GGTGGTCCAGGGTTTCTTA-3’	
Pik3c2b-forward	23	5’-CCGACTGGCTACAAAAACACAAC-3’	80
Pik3c2b -reverse	24	5’-GCCAGCACAAGAGTAGATGAAGTT-3’	

Supplementary Table 5

Antibodies used for semi-quantitative analysis of proteins by western-blotting

target protein	host	clonality	source	reference	clone[a]
BIN1 (pan-isoform)	mouse	monoclonal	Sigma	05-449	99D
BIN1 (exon11)	rabbit	polyclonal	IGBMC[b]	#2406	n/a
DNM2	rabbit	polyclonal	IGBMC	#2865	n/a
desmin	mouse	monoclonal	Dako	M0760	D33
DHPR	mouse	monoclonal	ABR[c]	MA3-920	1A
dysferlin	mouse	monoclonal	NCL[d]	NCL-Hamlet	Hamlet-1
ERα	rabbit	polyclonal	SCBT[e]	H-184	n/a
ERβ	rabbit	monoclonal	Abcam	ab92306	EPR3777
GAPDH	mouse	monoclonal	Merk	MAB374	6C5
myotubularin	rabbit	polyclonal	IGMBC	#2827	n/a
PIK3C2B	rabbit	polyclonal	BD bio[f]	611342	n/a
RyR1	mouse	monoclonal	DSHB[g]	AB_528457	34C

a: when appropriate the clone number is given
b: Antibody facility of the IGBMC, Illkirch, France
c: Affinity bioreagents, now part of ThermoFisher Scientific
d: Novocastra laboratories, now part of Leica Biosystems
e: Santa Cruz Biotechnologies
f : BD biosciences
g: Developmental Studies Hybridoma Bank, University of Iowa, Iowa City, IA, USA

Supplementary Table 6

Characteristics of human muscle cell lines

line code	line name	muscle	donor age	mutation	phenotype
Healthy-1	1235TE	*quadriceps*	44 years	none	none
Healthy-2	375/04	n/a	n/a	none	none
Healthy-3*	MX01209MBS	*vastus lateralis*	n/a	none	none
Healthy-4*	MX01409MBS	*tensor fascia lata*	n/a	none	none
XLMTM-1	493/03	n/a	n/a	n/a	n/a
XLMTM-2*	EG84 CLON 1	n/a	3 months	frameshift 238	severe
XLMTM-3	DM92	n/a	fetus	R474X	severe
XLMTM-4	T732 + 2A	*quadriceps*	fetus	T732 + 2A	severe

The table shows the main characteristics of the human muscle cell lines used in this study. Whenever available, the muscle from which the line was established, the age of the donor, the *MTM1* mutation and the phenotype is indicated. The lines marked with an asterisk were clonal expansions from purified myoblasts. The other lines are mixed cultures of myoblasts and other cell types, essentially muscle fibroblasts.

Supplementary Table 7

Primers used for quantitative analysis of human transcripts by RT-PCR

primer	exon	sequence	amplicon size (bp)
MTM1-forward	5	5'-GAGGCGCGACAAGTAGAGG-3'	137
MTM1-reverse	6	5'-AAACGCGTATCTCGTGAGGA-3'	
*BIN1*exon11-forward	10	5'-GAGCAAGCTCAACCAGAACC-3'	116
*BIN1*exon11-reverse	11	5'-GCCGCGAAAACAGTTTACTT-3'	
*BIN1*pan-forward	5	5'- ACAACGACCTGCTGTGGATGG -3'	100
*BIN1*pan-reverse	5/6	5'- CGTGACTTGATGTCGGGGAACT -3'	
DNM2-forward	6	5'-CATGGGCACGCCACATCT-3'	211
DNM2-reverse	8	5'-CTCAAAATCCACCCCAAACT-3'	
GAPDH-forward	7	5'-CTGCACCACCAACTGCTTAG-3'	107
GAPDH-reverse	8	5'-TCTTCTGGGTGGCAGTGATG-3'	

Supplementary Methods

▪ Quantification of tamoxifen and its metabolites

The concentrations of the TAM stereoisomers (Z)-TAM (the clinically active drug given to the mice) and (E)-TAM, and the Z and E stereoisomers of the TAM metabolites 4-hydroxytamoxifen (OHT), N-desmethyl-TAM (NDT) and 4-hydroxy-N-desmethyl-TAM (endoxifen; ENDO) were determined by ultra-performance liquid chromatography-tandem mass spectrometry in the plasma as well as in the leg muscle bulk of TAM-treated mice as described[2].

Briefly, the muscle bulk was pulverized in liquid nitrogen-cooled mortars. Twenty milligrams of the muscle powder was homogenized for 30 seconds with a hand-held tissue homogenizer (Omni International, Kennesaw, GA) in a mixture composed of 900 µL of absolute ethanol and 100 µL of deuterated internal standards solution (25-50 ng.mL^{-1} of 1:1 E/Z mixtures in methanol). The tissue homogenate was then centrifuged (4°C for 10 minutes at 16000xg). Seven hundred µl of the supernatant fluid was transferred into a propylene tube and dried under nitrogen at room temperature. The residue was reconstituted in 100 µL of acetonitrile, vortex-mixed, diluted with 200 µL of a buffer solution (10 mM ammonium formate, containing 0.25% formic acid) and centrifuged again as above. Supernatant fluid (150 µL) was introduced in a high-performance liquid chromatography glass microvial, and 20 µL was injected into the high-performance liquid chromatography system. Ultra-performance liquid chromatography-tandem mass spectrometry conditions (mobile phases, elution gradient, and mass spectrometer conditions) were identical for assaying both plasma and tissue levels. Calibration curves for tissue samples, prepared in ethanolic matrix (20 mg.mL^{-1}), ranged from 0.05 to 3 ng mL^{-1} for (E)- ENDO, 0.025 to 3 ng mL^{-1} for (Z)-ENDO, and 0.013 to 3 ng/mL for (Z)-OHT, (Z)-NDT, and (Z)-TAM. In this specific setting, the method was precise and accurate with the inter-assay precision (CV %) and accuracy (bias %) ranging between 1% and 13% and 8.9% and 6.1%, respectively. For plasma and muscle sample, (E)-TAM, (E)-NDT, and (E)-OHT levels were quantified with the calibration curves of their corresponding Z isomers. In plasma and tissue samples, E-TAM isomer was chromatographically identified by comparison of its retention time with that of the purchased pure standard (Toronto Research Chemicals Inc., North York, ON, Canada). (E)-NDT and (E)-OHT isomers were tentatively identified by comparison of their retention times with those of E isomers produced in vitro by exposing methanolic solutions of the corresponding Z isomers to UV light (254 nm) for 3 hours. The results are expressed as ng.mL^{-1} of plasma, ng.g^{-1} of tissue.

▪ Fiber typing

Fiber typing was performed by immunohistochemistry using mouse monoclonal antibodies against specific myosin heavy chains (MyHCs), according to standard procedures as described previously[2,7]. In brief, transverse sections prepared in the TA were incubated (1 µg.mL^{-1}) with one of the primary monoclonal antibodies BA-D5, SC-71, BF-35, and BF-F3 (Developmental Studies Hybridoma Bank, Iowa City, IA) against MyHCs type 1, 2a, all but 2x, and 2b fibers, respectively. BA-D5, SC-71, and BF-35 antibodies were detected with a goat anti-mouse IgG antibody conjugated to Alexa Fluor 594 (Molecular Probes) (1 µg.mL^{-1}), and the connective tissue was counterstained with wheat germ agglutinin (WGA) conjugated to AF488 (2 µg.mL^{-1}). The BF-F3 antibody was detected with a goat anti-mouse IgM antibody conjugated to Alexa Fluor 488 (Molecular Probes) (2 µg.mL^{-1}) and the connective tissue was counterstained with WGA-AF594 (Molecular Probes) (2 µg.mL^{-1}). The fibers stained positive for BA-D5, SC-71 and BF-F3 were classified as type I, IIA and IIB, respectively; the negative fibers upon BF-35 staining were classified as IIX.

▪ Molecular effects of tamoxifen on human muscle cell lines

Human muscle cell lines (4 from healthy donors and 4 from XLMTM individuals; see Supplementary Table 6), some of which were described elsewhere[14,15], were acclimatized over 2 passages to a growth medium made of DMEM:Ham's F10 (1:1) supplemented with 20% fetal bovine serum, ciprofloxacin (10 µg.mL^{-1}) and FGF-2 (5 ng.mL^{-1}). For experiments, cells were trypsinized and plated on gelatin-coated 6-well plates (Falcon, BD Biosciences). Myogenic differentiation was induced by switching sub-confluent cultures to a medium made of DMEM, 2% horse serum and ciprofloxacin. When indicated, the cultures were exposed to tamoxifen from differentiation days 2 to 7. Cell extracts were prepared using RNeasy Plus mini kit (Qiagen) or RIPA buffer for qRT-PCR and western blotting respectively, which were performed essentially as described above. Primers specific to human transcripts are shown in Supplementary Table 7.

Supplementary References

1. Buj-Bello, A., *et al.* The lipid phosphatase myotubularin is essential for skeletal muscle maintenance but not for myogenesis in mice. *Proc. Natl. Acad. Sci. USA* **99**, 15060-15065 (2002).
2. Dorchies, O.M., *et al.* The anticancer drug tamoxifen counteracts the pathology in a mouse model of duchenne muscular dystrophy. *Am. J. Pathol.* **182**, 485-504 (2013).
3. Dorchies, O.M., *et al.* Green tea extract and its major polyphenol (-)-epigallocatechin gallate improve muscle function in a mouse model for Duchenne muscular dystrophy. *Am. J. Physiol. Cell Physiol.* **290**, C616-625 (2006).
4. Hibaoui, Y., Reutenauer-Patte, J., Patthey-Vuadens, O., Ruegg, U.T. & Dorchies, O.M. Melatonin improves muscle function of the dystrophic mdx^{5Cv} mouse, a model for Duchenne muscular dystrophy. *J. Pineal. Res.* **51**, 163-171 (2011).
5. Reutenauer, J., Dorchies, O.M., Patthey-Vuadens, O., Vuagniaux, G. & Ruegg, U.T. Investigation of Debio 025, a cyclophilin inhibitor, in the dystrophic mdx mouse, a model for Duchenne muscular dystrophy. *Br. J. Pharmacol.* **155**, 574-584 (2008).
6. Reutenauer-Patte, J., Boittin, F.-X., Patthey-Vuadens, O., Ruegg, U.T. & Dorchies, O.M. Urocortins improve dystrophic skeletal muscle structure and function through both PKA- and Epac-dependent pathways. *Am. J. Pathol.* **180**, 749-762 (2012).
7. De Andrade, P.B.M., *et al.* Caloric restriction induces energy-sparing alterations in skeletal muscle contraction, fiber composition and local thyroid hormone metabolism that persist during catch-up fat upon refeeding. *Front. Physiol.* **6**, 254 (2015).
8. Cowling, B.S., *et al.* Amphiphysin (BIN1) negatively regulates dynamin 2 for normal muscle maturation. *J. Clin. Invest.* **127**, 4477-4487 (2017).
9. Tasfaout, H., *et al.* Antisense oligonucleotide-mediated Dnm2 knockdown prevents and reverts myotubular myopathy in mice. *Nat. Commun.* **8**, 15661 (2017).
10. Cowling, B.S., *et al.* Reducing dynamin 2 expression rescues X-linked centronuclear myopathy. *J. Clin. Invest.* **124**, 1350-1363 (2014).
11. Cowling, B.S., *et al.* Increased expression of wild-type or a centronuclear myopathy mutant of dynamin 2 in skeletal muscle of adult mice leads to structural defects and muscle weakness. *Am. J. Pathol.* **178**, 2224-2235 (2011).
12. Amoasii, L., *et al.* Phosphatase-dead myotubularin ameliorates X-linked centronuclear myopathy phenotypes in mice. *PLoS Genet.* **8**, e1002965 (2012).
13. Boittin, F.-X., *et al.* Ca^{2+}-independent phospholipase A_2 enhances store-operated Ca^{2+} entry in dystrophic skeletal muscle fibers. *J. Cell Sci.* **119**, 3733-3742 (2006).
14. Dorchies, O.M., *et al.* Normal innervation and differentiation of X-linked myotubular myopathy muscle cells in a nerve-muscle coculture system. *Neuromuscul. Disord.* **11**, 736-746 (2001).
15. Laporte, J., Kress, W. & Mandel, J.L. Diagnosis of X-linked myotubular myopathy by detection of myotubularin. *Ann. Neurol.* **50**, 42-46 (2001).
