# Supplementary material for: Tamoxifen prolongs survival and alleviates symptoms in mice with fatal X-linked myotubular myopathy
Source: Nat Commun. 2018 Nov 19;9:4848. doi: 10.1038/s41467-018-07058-4 (PMC6243013; doi:10.1038/s41467-018-07058-4)
Supplement: Supplementary file 7 — Description of Additional Supplementary Files [file 41467_2018_7058_MOESM7_ESM.docx]

**Title:** Supplementary Movie 1
**Description:** Activity and hind paws positioning in young (D40) untreated and tamoxifen-treated mice Wild type (WT) and Mtm-/y (Mtm1-null) mice were fed either a control diet or a tamoxifensupplemented diet (30 mg per kg of diet) from weaning (at D23) onward. The movie shows the exploratory and locomotor behaviors of a tamoxifen-treated WT mouse, an untreated Mtm1-null mouse, and a tamoxifen-treated Mtm1-null mouse at D40. Note that the untreated Mtm1-null mouse displays overt signs of hindlimb weakness and limited movement amplitude of the hind paws. Most of its weight is being placed on the knees. In contrast, in spite of a waddling gait, the tamoxifen-treated Mtm1-null mouse is more active and places its hind paws in a correct position when exploring its new environment.

**Title:** Supplementary Movie 2
**Description:** Activity and hind paws positioning in adult (D85) tamoxifen-treated Mtm1-null mice Wild type (WT) and Mtm-/y (Mtm1-null) mice were fed either a control diet or a tamoxifensupplemented diet (30 mg per kg of diet) from weaning (at D23) onward. The movie shows the exploratory and locomotor behaviors of a tamoxifen-treated WT mouse and two tamoxifentreated Mtm1-null mice at D85. At that age all the untreated animals have already succumbed to the disease (median survival was 45 days). Note that in spite of a variable phenotypic rescue, both tamoxifen-treated Mtm1-null mice were active, showed multiple rearing, and retained quite normal positioning of the hind paws. At around 13-14 sec, note the tamoxifentreated Mtm1-null mouse running in parallel to the tamoxifen-treated WT mouse.

**Title:** Supplementary Movie 3
**Description:** Activity and hind paws positioning in adult (D113) tamoxifen-treated Mtm1-null mice Wild type (WT) and Mtm-/y (Mtm1-null) mice were fed either a control diet or a tamoxifensupplemented diet (30 mg per kg of diet) from weaning (at D23) onward. The movie shows the exploratory and locomotor behaviors of a tamoxifen-treated WT mouse and two tamoxifentreated Mtm1-null mice at D113. At that age, all the untreated animals have already succumbed to the disease much earlier (median survival was 45 days) and tamoxifen increased the lifespan 2.5 times. Note that the waddling gait already observed at D40 (see Supplementary Movie 1) persists but is not significantly aggravated over time. In spite of evident weakness in the hindlimbs, the tamoxifen-treated mice were actively exploring the new environment they were placed in and were able to walk on their hind paws, demonstrating much delayed disease progression.

**Title:** Supplementary Movie 4
**Description:** Rearing and climbing behavior in an adult (D113) tamoxifen-treated Mtm1-null mouse Wild type (WT) and Mtm-/y (Mtm1-null) mice were fed either a control diet or a tamoxifensupplemented diet (30 mg per kg of diet) from weaning (at D23) onward. The movie shows a tamoxifen-treated Mtm1-null mouse at D113 rearing multiple times before raising its own body above an object (Mouse House). At that age, all the untreated animals have already succumbed to the disease much earlier (median survival was 45 days) and tamoxifen increased the lifespan 2.5 times. Note that the mouse was placing its hind paws in a normal position and was able to use both fore paws and hind paws to push its body upward. Also note the preserved mobility of the toes, of both the lower and upper limbs, of the trunk and of the neck.
