# Supplementary material for: Tamoxifen prolongs survival and alleviates symptoms in mice with fatal X-linked myotubular myopathy
Source: Nat Commun. 2018 Nov 19;9:4848. doi: 10.1038/s41467-018-07058-4 (PMC6243013; doi:10.1038/s41467-018-07058-4)
Supplement: Supplementary file 2 — Peer Review File [file 41467_2018_7058_MOESM2_ESM.pdf]

This manuscript has been previously reviewed at another journal that is not operating a transparent peer review scheme. The manuscript was considered suitable for publication without further review at Nature Communications.
